# The C-Mannosylome of Human Induced Pluripotent Stem Cells Implies a Role for ADAMTS16 C-Mannosylation in Eye Development

**DOI:** 10.1016/j.mcpro.2021.100092

**Published:** 2021-05-08

**Authors:** Karsten Cirksena, Hermann J. Hütte, Aleksandra Shcherbakova, Thomas Thumberger, Roman Sakson, Stefan Weiss, Lars Riff Jensen, Alina Friedrich, Daniel Todt, Andreas W. Kuss, Thomas Ruppert, Joachim Wittbrodt, Hans Bakker, Falk F.R. Buettner

**Affiliations:** 1 Institute of Clinical Biochemistry, Hannover Medical School, Hannover, Germany; 2 Centre for Organismal Studies Heidelberg, Heidelberg University, Heidelberg, Germany; 3 Zentrum für Molekulare Biologie der Universität Heidelberg (ZMBH), DKFZ-ZMBH Alliance, Heidelberg, Germany; 4 HBIGS, Heidelberg Biosciences International Graduate School, Heidelberg University, Heidelberg, Germany; 5 Leibniz-Institut für Analytische Wissenschaften-ISAS-e.V., Dortmund, Germany; 6 Human Molecular Genetics Group, Department of Functional Genomics, Interfaculty Institute for Genetics and Functional Genomics, University Medicine Greifswald, Greifswald, Germany; 7 Department for Molecular and Medical Virology, Ruhr University Bochum, Bochum, Germany; 8 European Virus Bioinformatics Center (EVBC), Jena, Germany

**Keywords:** C-mannosylation, human induced pluripotent stem cells, secretomics, ADAMTS16, coloboma, ACN, acetonitrile, CBiPSC2, human cord blood–derived induced pluripotent stem cell clone 2, CHO, Chinese hamster ovary, eGFP, enhanced GFP, EIC, extracted ion chromatogram, ESI, electrospray ionization, FA, formic acid, FCS, fetal calf serum, HEK, human embryonic kidney, hiPSC, human induced pluripotent stem cell, LFQ, label-free quantification, LTQ, Linear Trap Quadrupole, MRM, multiple reaction monitoring, RI, ROCK inhibitor, RT, retention time, sgRNA, single-guide RNA, TFA, trifluoroacetic acid, THBS1, thrombospondin 1, TSR, thrombospondin type 1 repeat

## Abstract

C-mannosylation is a modification of tryptophan residues with a single mannose and can affect protein folding, secretion, and/or function. To date, only a few proteins have been demonstrated to be C-mannosylated, and studies that globally assess protein C-mannosylation are scarce. To interrogate the C-mannosylome of human induced pluripotent stem cells, we compared the secretomes of CRISPR–Cas9 mutants lacking either the C-mannosyltransferase DPY19L1 or DPY19L3 to WT human induced pluripotent stem cells using MS-based quantitative proteomics. The secretion of numerous proteins was reduced in these mutants, including that of *A D*isintegrin *A*nd *M*etalloproteinase with *T*hrombo*S*pondin Motifs 16 (ADAMTS16), an extracellular protease that was previously reported to be essential for optic fissure fusion in zebrafish eye development. To test the functional relevance of this observation, we targeted *dpy19l1* or *dpy19l3* in embryos of the Japanese rice fish medaka (*Oryzias latipes*) by CRISPR–Cas9. We observed that targeting of *dpy19l3* partially caused defects in optic fissure fusion, called coloboma. We further showed in a cellular model that DPY19L1 and DPY19L3 mediate C-mannosylation of a recombinantly expressed thrombospondin type 1 repeat of ADAMTS16 and thereby support its secretion. Taken together, our findings imply that DPY19L3-mediated C-mannosylation is involved in eye development by assisting secretion of the extracellular protease ADAMTS16.

## Highlights

• TSR1 of ADAMTS16 can be C-mannosylated.
• Deletion of DPY19L1 or DPY19L3 in hiPSCs caused reduced secretion of ADAMTS16.
• Targeting of *dpy19l3* in medaka occasionally led to coloboma.

## Graphical Abstract

**Figure:**
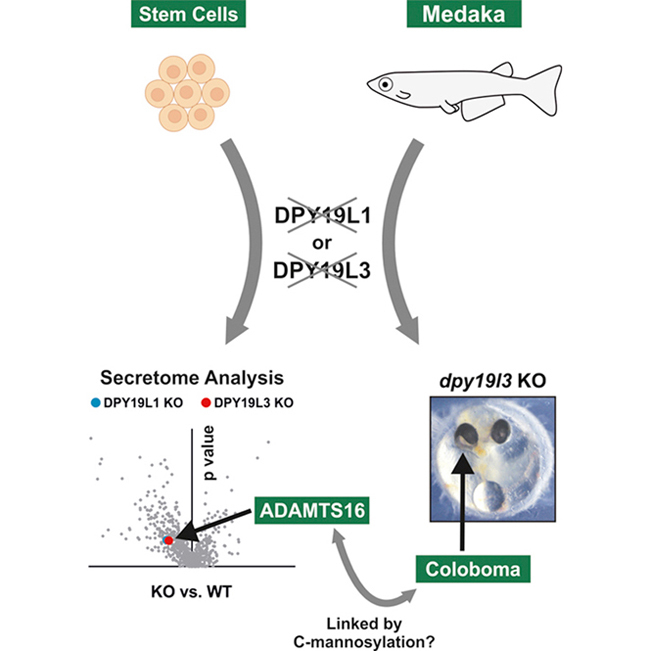

## In Brief

We identified ADAMTS16 as a target protein for C-mannosylation and showed that this modification is needed for proper secretion of ADAMTS16. Targeting a distinct C-mannosyltransferase by CRISPR–Cas9 in medaka fish embryos caused defects in eye development. We conclude that these developmental defects are caused by reduced secretion of ADAMTS16 when C-mannosylation is missing.

C-mannosylation, in addition to N-glycosylation and O-glycosylation, is a further but less well-studied type of protein glycosylation taking place in the endoplasmic reticulum (1, 2). In C-mannosylation, the C1 atom of an α-mannose is attached *via* a carbon–carbon bond to the indole C2 atom of a tryptophan residue typically located in the protein consensus sequence WxxW/C (3, 4, 5). We uncovered the genetic basis of C-mannosylation by identification and functional characterization of the C-mannosyltransferase of *Caenorhabditis* (*C*.) *elegans* (6). This discovery enabled specific deletion of its mammalian homologs, which revealed that DPY19L1 and DPY19L3 are C-mannosyltransferases with distinct specificities for the first tryptophan in WxxW and WxxC motifs within thrombospondin type 1 repeats (TSRs), respectively (7). About 18% of all human transcripts with signal peptides and/or transmembrane helices were predicted to contain C-mannosylation sites (8). However, so far, C-mannosylation has only been shown for a few proteins, which mostly belong to the TSR superfamily. A TSR is an independently folding structure of about 60 amino acids that typically contains six conserved cysteine residues and a conserved WxxWxxWxxC motif of which all tryptophans can be C-mannosylated (9, 10). All 19 members of the ADAMTS (*A D*isintegrin *A*nd *M*etalloproteinase with *T*hrombo*S*pondin motifs) superfamily and the six human ADAMTS-like proteins contain one or more TSRs (11). ADAMTS proteins are large secreted metalloproteinases with pleiotropic roles in tissue and extracellular matrix morphogenesis and remodeling (12). C-mannosylation has been shown for ADAMTS20, ADAMTS13, and ADAMTS5 (13, 14, 15), but data on the functional importance of C-mannosylation for that large protein family remain elusive.

The tryptophan-bound mannose of TSRs has a variable conformation (16) and potentially adapts a structure supporting protein folding and stability (17). Accordingly, cell surface expression or secretion of several proteins has been shown to depend on C-mannosylation (6, 7, 18, 19, 20, 21, 22, 23, 24, 25).

Acknowledging the importance of C-mannosylation for protein secretion and motivated by an interest in the functional role of C-mannosylation for early developmental processes, we quantitatively compared the levels of secreted proteins from cell culture supernatants of human induced pluripotent stem cells (hiPSCs) and derived KOs of the C-mannosyltransferases DPY19L1 or DPY19L3 by MS. In a complementary study, we applied targeted genome editing on *dpy19l1* and *dpy19l3* in the Japanese rice fish medaka (*Oryzias latipes*). The combined analysis of our cellular and organismal studies uncovered the importance of C-mannosylation for secretion and function of ADAMTS16.

## Experimental Procedures

### Maintenance of Mammalian Cells

All cells were maintained at 37 °C, 5% CO_2_, and 85% relative humidity in cell culture vessels purchased from Greiner Bio-One. Cell culture reagents were purchased from Thermo Fisher Scientific unless otherwise stated. All stem cell–based experiments were performed as described previously (26) with the hiPSC line CBiPSC2 (human cord blood–derived induced pluripotent stem cell clone 2 (27)) and genetically modified derivatives thereof. In brief, cells were maintained as colonies in 6-well plates on γ-irradiated mouse embryonic fibroblasts (EmbryoMax PMEF-P3, strain CF-1; Millipore) in stem cell medium (KO Dulbecco's modified Eagle's medium [DMEM], 20% [v/v] KO serum replacement, 1% [v/v] minimum essential medium nonessential amino acids, 0.5% [v/v] GlutaMAX, and 0.1 mM 2-mercaptoethanol supplemented with 50 ng/ml basic fibroblast growth factor [Institute of Technical Chemistry]) and passaged every 3 to 4 days by collagenase IV treatment. For culture under feeder-free conditions, cells were grown on Matrigel (Corning; 1:60 dilution) in mTeSR1 (STEMCELL Technologies) and passaged every 3 to 4 days by treatment with Dispase (STEMCELL Technologies). Human embryonic kidney (HEK) 293T and Chinese hamster ovary (CHO)-K1 cells were grown in DMEM/Ham's F-12 (Biochrom) with 10% (v/v) and 5% fetal calf serum (FCS) (Biochrom), respectively, and passaged once a week using trypsin–EDTA.

### Generation of DPY19L1 KO and DPY19L3 KO Cells

For genome editing in stem cells, we applied the CRISPR–Cas9 system each with two different single-guide RNAs (sgRNAs) to mediate macrodeletions in DPY19L1 and DPY19L3, respectively. The CCTop algorithm (28) was applied to screen DPY19L1 and DPY19L3 for appropriate target sites ending up with the following (protospacer adjacent motifs are underlined): 5’-GAGGTGTGTTATATGGCTCCTGG and 5’-GCTGATGGCTAGGCTATAGGAGG (DPY19L1); 5’-GATAATCTAGGTACAATTGGTGG and 5’-GAGCTGTGACATAGATCGCCTGG (DPY19L3). sgRNA templates were individually cloned into a plasmid coding for a SpCas9 endonuclease and enhanced GFP (eGFP) (29). hiPSCs from feeder-free culture were singularized by TrypLE, and 1 × 10^6^ cells were cotransfected with 5 μg of each of the two sgRNA coding plasmids using a NEON transfection device (Thermo Fisher Scientific) with 1200 V for 2 × 20 ms. Cells were reseeded in mTeSR1 medium supplemented with 10 μM Rho-associated coiled-coil containing protein kinase inhibitor (ROCK inhibitor [RI]; Stem Cell Technologies; Y-27632), and eGFP-positive cells were sorted after 3 days applying fluorescence-activated cell sorting. About 5 × 10^3^ sorted cells were seeded on a murine embryonic fibroblast–coated 10 cm dish in stem cell medium supplemented with 10 μM RI and cultured for 10 days until separate single-cell colonies became visible. Colonies were picked manually by using 200 μl pipette tips and under a light microscope at sterile conditions. Picked colonies were transferred into murine embryonic fibroblast–coated 48-well plates in stem cell medium supplemented with 10 μM RI. After 4 days, single-cell clones were passaged, and cells were extracted for direct cell lysis and genotyping by PCR analysis (supplemental Fig. S1*B*).

### RNA-seq Analysis of WT, DPY19L1 KO, and DPY19L3 KO hiPSCs

For total RNA extraction, NucleoSpin RNA Kit (Macherey–Nagel) was applied. Samples were sequenced on an Ion Torrent S5 XL Instrument (Thermo Fisher Scientific). Read mapping was carried out with STAR, version 2.6.0 with default parameters and the GRCh37 release 87 reference genome (30). HTSeq, version 0.10.0, with default parameters was used to determine read counts per gene (31). Statistical analysis was performed with R (see Experimental Design and Statistical Rationale section for details).

### Transient Protein Expression

To achieve appropriate transient protein expression in diverse mammalian cells, the eGFP of the pCAG–eGFP vector (Addgene plasmid #89684) was excised (XhoI and BglII) and replaced by a section of pSecTagB (Invitrogen) comprising the signal peptide for the secretory pathways, multiple cloning site, c-*myc* epitope, and polyhistidine tag. This resulted in the *pCAG-SecTagB* vector. Protein coding sequences were amplified from hiPSC-derived complementary DNA and cloned into KpnI and NotI sites of the *pCAG-SecTagB* vector (supplemental Fig. S2). For protein expression in stem cells, 1 × 10^7^ hiPSCs were transfected with 50 μg of plasmid (split into four transfections with each 2.5 × 10^6^ cells and 12.5 μg plasmid) using a NEON transfection device (Thermo Fisher Scientific) with 1200 V for 2 × 20 ms. Cells were reseeded in four T25 cell culture flasks on Matrigel in TeSR–E8 medium supplemented with RI. For protein expression in HEK 293T and CHO-K1 cells, 3 × 10^6^ cells were seeded into a T75 cell culture flask in 20 ml DMEM/Ham's F-12 + 10% FCS or 5% FCS, respectively. For protein expression in KO cells, two approaches were pooled in the end. Cells were transfected after 24 h with 20 μg plasmid using 100 μg polyethyleneimine in 2 ml Opti-minimum essential medium followed by medium exchange after additional 6 h. For coexpression with a construct encoding myc-tagged epidermal growth factor repeats 9–14 of mouse Notch1, cells were cotransfected with additional 5 μg of the respective plasmid using 125 μg polyethyleneimine. Secreted proteins were purified from the medium after 3 days by applying nickel affinity chromatography and SDS-PAGE as described (7). The medium was adjusted to a final concentration of 500 mM NaCl, 20 mM Tris–HCl, pH 8, and 20 mM imidazole (washing buffer concentration) and filtered through a 0.2 μm membrane (Millipore). The medium was applied to a 1 ml HisTrap HP column (GE Healthcare) followed by a 10 ml washing step with washing buffer. To elute proteins from the column, the imidazole concentration was increased from 20 to 500 mM in 7 ml. After protein precipitation with acetone, proteins were resuspended in Laemmli buffer containing 5% (v/v) 2-mercaptoethanol and incubated at 95 °C for 5 min. Proteins were further purified by SDS-PAGE (15% [w/v]), stained with Coomassie, cut out, and digested with Trypsin Gold (Promega, specifically cleaving C-terminal to lysine and arginine residues) or AspN (Promega, specifically cleaving N-terminal to aspartic acid, and, to a lesser extent, glutamic acid residues) overnight at 37 °C. In a separate gel, purification of proteins was confirmed by Western blot using mouse anti-myc 9E10 antibody and IRDye 800–conjugated goat anti-mouse antibody (LI-COR). Quantification of proteins detected by Western blot analysis was performed with Image Studio 4 (LI-COR).

### MS Analysis of Recombinantly Expressed Proteins

Proteolytically digested proteins were analyzed using an electrospray ionization (ESI) quadrupole-TOF (Q-TOF) Ultima mass spectrometer (Waters) coupled with a nanoACQUITY UPLC System with an analytical C18 column (Waters, BEH130 C18, 100 × 100 μm, 1.7 μm particle size). The MS analysis was performed as described previously (6). LC was performed at a flow rate of 0.3 μl/min for 45 min starting with buffer A (water, 0.1% [v/v] formic acid [FA]) for 0.33 min followed by a linear gradient of buffer B (acetonitrile [ACN], 0.1% [v/v] FA) from 1% to 35% within 29.67 min. Buffer B was increased linearly from 35% to 85% within 1 min followed by 85% buffer B for 1 min. Subsequently, buffer B was decreased to 1% within 0.1 min and the system was washed with 99% buffer A for 12.9 min. Spectra were recorded in positive ion mode. Peptides were either recorded in MS mode or automatically subjected to fragmentation. The MassLynx V4.1 software (Waters) was applied to analyze spectra. For extracted ion chromatograms (EICs), the mass chromatogram function of MassLynx (mass error set to 0.3 Da) was applied to search for the masses corresponding to glycosylated and nonglycosylated peptides within the MS1 chromatograms. Fragment ion masses were calculated with the web-based *Fragment Ion Calculator* (Institute for Systems Biology) and annotated manually in detected MS/MS spectra. In addition, MS spectra were automatically searched with ProteinLynx V2.2.5 software (Waters) against custom-made databases containing sequences of individual proteins using preconfigured settings except “peptide tolerance” was set to 0.3 Da and “validation of results” was switched off. Oxidation (M), C-mannosylation (W), and modification by fucose or fucose–glucose (T, for thrombospondin 1 [THBS1] samples only) were chosen as variable modifications and carbamidomethyl (C) as fixed modification.

### Sample Preparation for Secretome Analysis

For the secretome analysis, hiPSCs were cultured under feeder-free conditions. About 100,000 to 400,000 cells, depending on the growth behavior of different clones, were seeded on a Matrigel-coated T25 cell culture flask in TeSR–E8 medium supplemented with RI. Medium was changed after 24 h, and cells were cultured in 4 ml TeSR–E8 medium for 72 h. Provided that a confluency of 40% to 60% was reached and the medium did not contain a large amount of detached and apoptotic cells, the cell culture supernatant was harvested and prepared for LC–MS analysis as described previously (32). Concisely, cell culture supernatant was centrifuged at 2000*g* for 5 min to remove cell debris. Proteins were precipitated applying the trichloroacetic acid/sodium lauroyl sarcosinate method (33), resuspended in 50 μl Laemmli buffer containing 5% (v/v) 2-mercaptoethanol, incubated at 95 °C for 5 min, and separated by SDS-PAGE followed by Coomassie staining. Each sample lane was cut in five to seven fractions, which were further cut into small pieces and digested with Trypsin Gold (Promega) overnight at 37 °C.

### MS Analysis of hiPSC Secretomes

MS analysis was performed applying a Linear Trap Quadrupole (LTQ) Orbitrap-Velos mass spectrometer coupled to a reversed-phase LC system as described previously (32). Reversed phase chromatography was performed by using a nanoflow ultrahigh pressure LC system (rapid separation LC; Thermo Fisher Scientific) with a trapping column (2 cm length, 75 μm inner diameter, and 3 μm C18 particle size) and a reversed-phase separating column (50 cm length, 75 μm inner diameter, and 2 μm C18 particle size). For trapping, a flow rate of 6 μl 0.1% trifluoroacetic acid (TFA)/min was applied for 5 min. For separation, flow rate was set to 250 nl/min at 45 °C and a gradient of buffer B (80% [v/v] ACN, 0.1% [v/v] FA) in buffer A (0.1% [v/v] FA) was applied. The concentration of buffer B was increased from 4% to 25% in 115 min, from 25% to 50% in 25 min, from 50% to 90% in 5 min, and hold at 90% for additional 10 min. Ionization and electrospray injection of peptides was performed with a Nano Spray Flex Ion Source II by using metal-coated fused silica emitters (Silica Tip, 20 μm inner diameter and 10 μm tip inner diameter) and a voltage of 1.2 kV. Overview scans were performed in the Orbitrap analyzer with a resolution of 60 k at *m*/*z* 400 in a mass range of *m*/*z* 300 to 1600 and stored in profile mode. The ten most intensive double-charged and triple-charged peptides with a minimum intensity of 2000 counts were selected for collision-induced dissociation fragmentation with normalized collision energy of 38, an activation time of 10 ms, and an activation Q of 0.250 in the LTQ. Recording of fragment ion mass spectra was performed at the LTQ with normal scan rate. Spectra were stored as centroid *m*/*z* value and intensity pairs. Peptides that were subjected to fragmentation were excluded within a time frame of 70 s and a mass window of 10 ppm of *m*/*z*.

### Processing of MS Raw Data by MaxQuant

MaxQuant software suite, version 1.6.11.0 (34), with the integrated search engine Andromeda was applied to process raw data for the identification and quantification of proteins. A combined analysis was performed for all 84 MS raw files. Unless stated otherwise, preconfigured standard settings were used: digest mode set to specific, missed cleavages set to 2, oxidation (M) and acetylation (N-term) chosen as variable modifications, carbamidomethyl (C) set as fixed modification, mass tolerance set to 20 ppm (precursor) and 0.5 Da (MS/MS), respectively. MS spectra were searched against the UniProt database of human proteins (reviewed + unreviewed, 188,357 entries, database downloaded on 09/03/2020) with a false discovery rate of 1% for proteins and peptides. Intensity-based absolute quantification and label-free quantification (LFQ) were chosen for LFQ of proteins with *LFQ min. ratio count* set to 1 and *Fast LFQ* switched off. The MaxQuant output file “proteinGroups.txt” is provided in supplemental Table S1.

### Secretome Data Analysis With Perseus Software

Perseus software, version 1.6.2.1 (35), with implemented annotations of the human proteome (mainPerseusAnnot.txt.gz downloaded on 02/04/2020 at http://annotations.perseus-framework.org) was used for filtering and statistical analysis (see Experimental Design and Statistical Rationale section for details) of the “protein groups” output table of the MaxQuant analysis. Proteins or protein groups identified by at least one peptide within any of all conditions were uploaded into a single matrix followed by exclusion of contaminants and proteins only identified by site or matching to the reverse database. Extracellular proteins were filtered by exclusion of protein IDs not annotated with Gene Ontology Cellular Compartment slim names “*extracellular matrix*,” “*extracellular region*,” or “*extracellular space*.” In addition, analysis was restricted to proteins identified by at least two different peptides and detected in at least two replicates of WT cells or both clones of the DPY19L1 KO or the DPY19L3 KO. For statistical analysis, LFQ values were normalized by transformation into the base-2 logarithm, and missing values were replaced by imputation of low LFQ values drawn from the normal distribution of the dataset (width: 0.3, down shift: 1.8, separately for each column). Levels of secreted proteins in different cell lines were compared by pairwise *t* tests. Proteins containing consensus sites for C-mannosylation were annotated by applying a custom-made R script searching all UniProt ID–related protein sequences of a protein group for the presence of WxxW or WxxC motifs. Fisher exact tests were applied to identify protein domains enriched within the group of proteins with altered abundance in the KO secretomes. Results were visualized by GraphPad Prism software (version 5).

### Experimental Design and Statistical Rationale

For both, RNA-Seq and secretomics, experiments were performed with WT hiPSCs (control) and two individual clones of both, DPY19L1 KO and DPY19L3 KO, to minimize clonal effects caused by genome editing and clone selection. Two biological replicates for RNA-Seq and three biological replicates for secretomics were analyzed for WT and each KO clone. For secretomics, all biological replicates of independent cultures of WT (n = 3) and of both clones for DPY19L1 KO (two clones with n = 3, each → n = 6) and DPY19L3 KO (two clones with n = 3 each → n = 6) were grouped, and the WT group was compared separately to the DPY19L1 KO and DPY19L3 KO group by pairwise *t* tests (two-tailed Student's *t* test with S0 = 0, no valid value filter, threshold *p* value = 0.05). Supplemental Figure S3 indicates normal distributions of all datasets. For enrichment analysis, Fisher exact tests (*p* = 0.05) were applied to identify enrichment of protein families (according to the *Pfam* database) among the 25 proteins that were significantly affected in the DPY19L1 KO or DPY19L3 KO groups and that contain at least one consensus site (WxxW/WxxC) for C-mannosylation, referred to the frequency within all 398 extracellular proteins that remained upon filtering. For RNA-Seq analyses, normalization and differential expression calculations were carried out using the R package DESeq (36). RNA-Seq analysis was performed with four biological replicates of WT, two biological replicates of DPY19L1 C1, DPY19L1 C2, and DPY19L3 C1 as well as one replicate of DPY19L3 C2 cells. For comparison of global gene expression, the biological replicates of WT and the KO clones were pooled separately. To analyze the effect of the DPY19L1 KO and the DPY19L3 on various target genes, the biological replicates of WT as well as all replicates of both clones of the each KO were pooled. Differential expression analysis was conducted for the WT *versus* KO conditions.

### Multiple Reaction Monitoring Assay Development and Analysis of Endogenous C-mannosylated THBS1

A THBS1 fragment comprising TSR1 to 3 was cloned directly into the HindIII and XbaI sites of pSecTagB (Invitrogen; supplemental Fig. S2), expressed in HEK 293T cells, purified, separated by SDS-PAGE, and digested with AspN. Peptides were resolubilized in 20% ACN/0.1% (v/v) TFA and incubated for 5 min at room temperature, then diluted 10-fold with 0.1% of TFA to gain a final concentration of 2% ACN. Stock solutions were stored at −20 °C. These peptides were used for multiple reaction monitoring (MRM) assay development according to tier 3 for exploratory studies (37). All possible transitions for four peptides of THBS1 within the 250 to 1250 *m*/*z* range, of which two contained consensus sites for C-mannosylation, were calculated using Skyline, version 20.1 (38) and monitored in an iterative empirical process as reviewed (39). All possible precursor ions with charges 2 to 4 and all b and y ions were measured using an unscheduled MRM method to determine most intense transitions and specific peptide retention times (RTs). Collision energies were manually optimized for individual transitions (38), and RTs were calibrated to a set of synthetic indexed RT peptides (40). LC for MRM analysis was performed on a Waters nanoACQUITY UPLC System equipped with a trapping column (Waters, Symmetry C18; 2 cm length, 180 μm inner diameter, 5 μm C18 particle size, and 100 Å pore size) and an analytical column (Waters, M-Class Peptide BEH C18; 25 cm length, 75 μm inner diameter, 1.7 μm C18 particle size, and 130 Å pore size). Samples were trapped for 7 min at a flow rate of 10 μl/min with 99.4% of buffer A (1% [v/v] ACN and 0.1% [v/v] FA) and 0.6% of buffer B (89.9% [v/v] ACN and 0.1% [v/v] FA) and separated using a reverse-phase C18 column with the analytical column temperature set at 60 °C and at a flow rate of 300 nl/min. Approximately 4 to 10 pmol per peptides were separated with a linear gradient of buffer B in buffer A from 3% to 37% B in 40 min, followed by washing and reconditioning of the column to 3% B. The nanoACQUITY UPLC System was coupled online by liquid junction to an ESI-QTrap 5500 *via* a NanoSpray III Source (both Sciex). Uncoated precut emitters (Silica Tip, 20 μm inner diameter, 10 μm tip inner diameter; New Objectives) and a voltage of approximately 2.6 kV were applied for ESI.

The secretome sample was obtained by precipitation of hiPSC culture supernatants that were separated by SDS-PAGE and digested with AspN as described previously. Only proteins migrating above the 70 kDa marker band were excised to remove low–molecular weight proteins. By spiking in the digested peptides of the recombinant THBS1 fragment into the secretome sample, we accounted for possible RT shifts or ion suppression effects caused by the specific matrix. After a blank run with 0.1% TFA, which ensured that no carryover signal was present, the secretome sample without recombinant spike-in was measured using scheduled MRM to detect endogenously C-mannosylated THBS1 peptides. For detection of endogenously C-mannosylated peptides, 25% of one secretome AspN digest was used for one LC–MRM analysis. Final scheduled MRM measurements were performed with 1.8 s target scan time and 300 s MRM detection window width. EICs were loaded into Skyline 20.1, and peak boundaries were manually inspected. Results were visualized using GraphPad Prism software (version 5).

### Medaka Experiments

#### Animal husbandry of medaka and ethics statement

Medaka (*O. latipes*) Cab strain used in this study were kept as closed stocks in accordance to Tierschutzgesetz §11, Abs. 1, Nr. 1 and with European Union animal welfare guidelines. Medaka experimentation was performed at the one-cell stage and analyzed at embryonic stages prior to stage 41, that is, prior to self-feeding and neither the loss of embryonic features and is thus not considered animal experimentation nor is an approval by an ethics committee required. Fish were maintained in a constant recirculating system at 28 °C on a 14 h light/10 h dark cycle (Zucht-und Haltungserlaubnis AZ35-9185.64/BH).

#### Designing and cloning of sgRNAs

sgRNAs were designed with CCTop and standard conditions (28). The following target sites were used (protospacer adjacent motif in brackets): dpy19l1_T1 (5’-TCTCACGTTACTTTTGCCGT[GGG]), dpy19l1_T2 (5’-AAAGTAACGTGAGACGACCG[GGG]), dpy19l3_T1 (5’-TGGAGCGTGAAATCTCCTTT[CGG]), and dpy19l3_T2 (5’-GTTACAGGCGCCTTCCATCC[AGG]). Cloning of sgRNA templates and *in vitro* transcription was performed as detailed previously (28).

#### Microinjection and genotyping

One-cell stage WT medaka (*O. latipes*) Cab strain zygotes were microinjected into the cytoplasm with 150 ng/μl *Cas9* mRNA, 15 ng/μl per sgRNA, and 10 ng/μl *GFP* or *mCherry* mRNA as injection tracer. Embryos were maintained in embryo-rearing medium (170 mM NaCl, 4 mM KCl, 2.7 mM CaCl_2_.2H_2_O, 6.6 mM MgSO_4_.7H_2_O, and 170 mM Hepes) at 28 °C after injections and screened for GFP or mCherry expression 1 day post injection with a Nikon SMZ18 binocular. GFP- or mCherry-positive crispants were raised at 28 °C and phenotyped. Subsequently, PCR genotyping was performed on extracted DNA of pooled and single embryos: specimens were ground and DNA was extracted using 50 μl extraction buffer (0.4 M Tris–HCl [pH 8.0], 5 mM EDTA [pH 8.0], 0.15 M NaCl, and 0.1% SDS in distilled water with 1 mg/ml Proteinase K [Roche; 20 mg/ml]). Genotyping PCR was performed with Q5 High-Fidelity DNA Polymerase (New England Biolabs) with 98 °C at an initial denaturation for 2 min, followed by 30 cycles of 98 °C denaturation for 20 s, 67 °C annealing for 30 s, and 72 °C extension for 25 s. The following primers were used: *dpy19l1*_forward 5’-CCTCTAGTGTTTCTGTCCCCC, *dpy19l1*_reverse 5’-AGCTAGCACGCTAAAACCACT, *dpy19l3*_forward 5’-CATGCTGAACTCCAAACGCC, and *dpy19l1*_reverse 5’-GGGTTTGTGGCCTTTTTGCT. Amplicons were analyzed by gel electrophoresis, followed by gel extraction (Analytik Jena following manufacturer's instructions) and sequencing (MWG Eurofins).

## Results

### Deletion of C-mannosyltransferases in hiPSCs

**Fig. 1:**
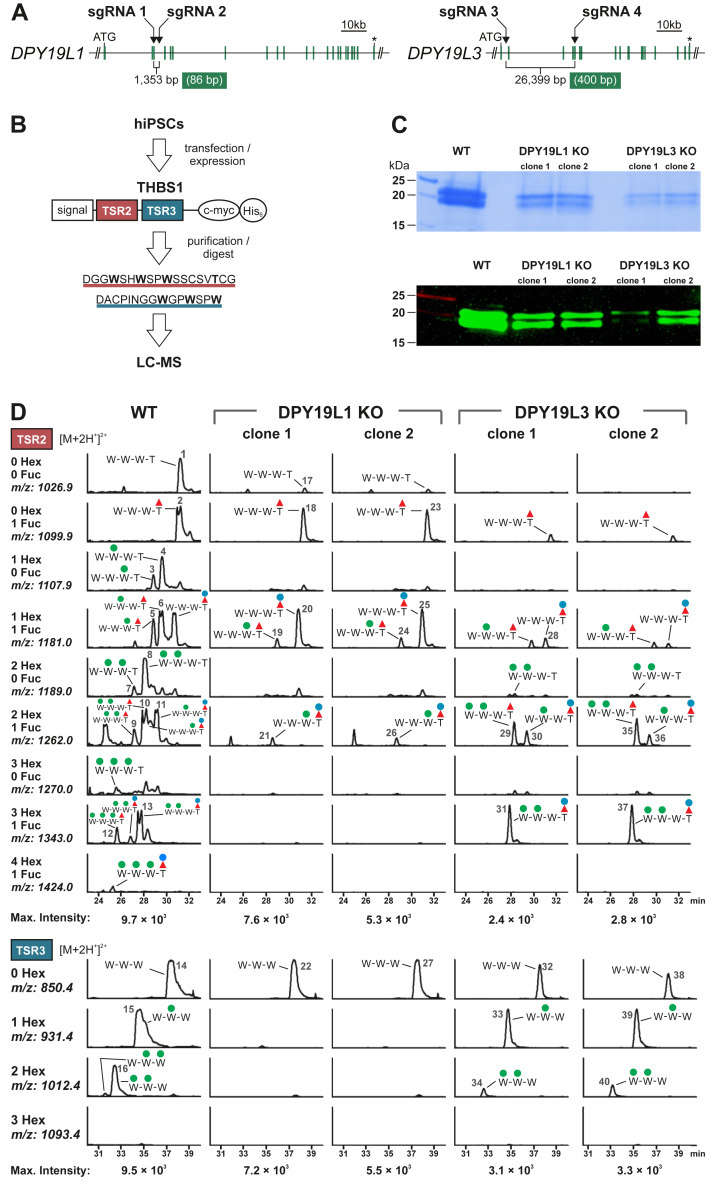
**Deletion of DPY19L1 and DPY19L3 in hiPSCs.***A*, schemes of *DPY19L1* and *DPY19L3* genes indicating exons (*green bars*), sgRNA positions, stop codons (*asterisks*), and the number of total or exonic (*green box*) deleted base pairs. *B*, scheme of the THBS1 fragment expressed in WT, DPY19L1 KO, and DPY19L3 KO hiPSCs. The fragment contains two TSRs (TSR2 and TSR3) resulting in the indicated peptides upon AspN digestion. *C*, SDS-PAGE analysis of a purified THBS1 fragment secreted from WT, DPY19L1 KO, and DPY19L3 KO hiPSCs. Coomassie staining (*upper panel*) and Western blot (*lower panel*, anti-myc). *D*, MS analysis of a purified and AspN-digested THBS1 fragment secreted from WT, DPY19L1 KO, or DPY19L3 KO hiPSCs. For each mutant, two individual clones were analyzed. Extracted ion chromatograms (EICs) of the TSR2-derived (*upper panels*) or TSR3-derived (*lower panels*) peptides ([M + H^+^]^2+^) with different numbers of hexoses (Hex) and fucoses (Fuc) are displayed. Corresponding spectra of each sample were adjusted to the intensity of the most intense glycoform (maximum intensity, as indicated at the *bottom*). Annotation of peaks was based on the parental ion mass, RT, and fragmentation spectra. Numbers 1 to 40 refer to the fragmentation spectra of the respective peaks provided in supplemental Figure S4. For each peptide, only tryptophan (W) and threonine (T) residues are depicted, with respective C-mannoses (*green circles*) or O-fucose (*red triangles*) ± glucose (*blue circles*). hiPSCs, human induced pluripotent stem cells; RT, retention time; sgRNA, single-guide RNA; THBS1, thrombospondin 1; TSR, thrombospondin type 1 repeat.

Two functional C-mannosyltransferases were reported for mammalian cells that are encoded by the genes *DPY19L1* and *DPY19L3* (7). To study C-mannosylation in human pluripotent stem cells, we aimed for the functional inactivation of DPY19L1 and DPY19L3, respectively, and excised parts of their coding sequence from the genome of the hiPSC line CBiPSC2 (27) by applying CRISPR–Cas9. We applied two different specific and high-scored sgRNAs (28) aiming to achieve large genomic deletions (macrodeletions) of 1353 bp for *DPY19L1* and 26,399 bp for *DPY19L3*, which, in addition, cause a frameshift (Fig. 1*A*). This strategy enabled simplified PCR-based screening of single cell–derived clones for genomic deletions (supplemental Fig. S1, *A* and *B*). For both C-mannosyltransferases, two individual KO clones were obtained (C1 and C2), one bearing a biallelic macrodeletion (DPY19L1 KO C1 and DPY19L3 KO C1) and one containing a monoallelic macrodeletion combined with insertions and deletions (indels) on the second allele resulting in a frameshift (DPY19L1 KO C2 and DPY19L3 KO C2) (supplemental Fig. S1, *C* and *D*).

Whole genome expression analysis by RNA-Seq of WT and KO hiPSCs revealed that their overall gene expression pattern including the expression of the stem cell markers *Klf4*, *Myc*, *Nanog*, *POU5F1*, and *Sox2* was similar. This suggests the absence of major genomic rearrangements or deleterious off-target effects that might have occurred during the procedure of gene editing (supplemental Fig. S1*E*).

To determine the effect of the genomic deletions in *DPY19L1* or *DPY19L3* on C-mannosylation, a C-terminally myc-His_6_-tagged construct comprising TSRs, TSR2, and TSR3, of human THBS1 with a cleavable N-terminal secretion sequence was expressed in WT and KO hiPSCs (Fig. 1*B*). Its purification from cell culture supernatants by nickel affinity chromatography and separation by SDS-PAGE indicated that DPY19L1- and DPY19L3-deficient hiPSCs secreted less of the THBS1 fragment than WT cells (Fig. 1*C*). This is in accordance with our previous observation that C-mannosylation was required for proper secretion of UNC5A TSRs (7). The THBS1 fragment was digested with AspN and analyzed by LC coupled to ESI tandem MS (LC–ESI–MS/MS). The resulting TSR2- and TSR3-derived peptides contained three putative C-mannosylation sites (W1, W2, and W3), each. The peptide originating from TSR2 in addition contains one O-fucosylation site. Theoretical *m*/*z* values of differentially glycosylated peptide species were searched in the obtained mass spectrometric data and displayed as EICs (Fig. 1*D*). Assignment of peaks in EICs was based on mass accuracy of the parental ion, peptide fragmentation spectra (supplemental Fig. S4) and RTs of peptides. Upon expression of the THBS1 fragment in WT hiPSCs, we detected the TSR2- and TSR3-derived peptides with the consensus site for C-mannosylation in their unmodified forms as well as with one or two mannoses. For the TSR2-derived peptide, an additional third mannose was observed. We further detected all respective non-, mono-, di-, and tri-C-mannosylated species of the TSR2-derived peptide with additional modification by O-fucose or O-fucose–glucose (Fig. 1*D* and supplemental Fig. S4). From the DPY19L1 KO clones, no peptides with C-Man on W1 or W2 could be detected and from DPY19L3 KO clones, and no peptides with C-mannosylation on W3 were detectable. This is in accordance to our previous findings that DPY19L1 is mainly acting on W1 and W2, whereas DPY19L3 acts on W3 of WxxWxxWxxC motifs of TSRs (7) and proves the functional inactivation of the respective C-mannosyltransferases in the hiPSC model (Fig. 1*D*).

### Quantitative Secretomics Revealed Potentially C-mannosylated Proteins

**Fig. 2:**
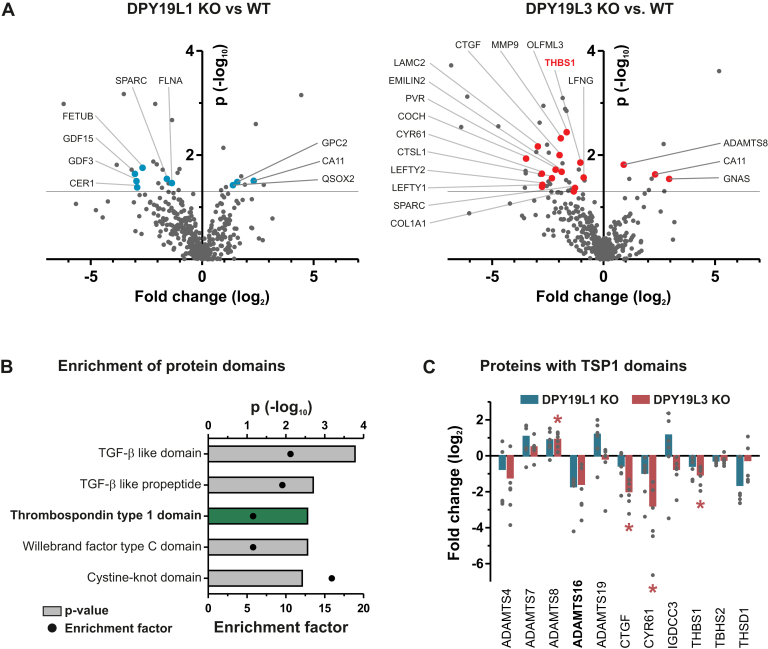
**TSR-containing proteins are reduced in cell culture supernatants of DPY19L1 KO and DPY19L3 KO hiPSCs**. *A*, volcano plots displaying ratios of protein levels (fold change) detected in cell culture supernatants for DPY19L1 KO *versus* WT (*left*) and DPY19L3 KO *versus* WT (*right*). Significantly affected proteins with consensus sites for C-mannosylation are highlighted by *blue* (DPY19L1 KO) and *red* (DPY19L3 KO) dots, respectively. *Dots* above the horizontal line represent statistically significantly (*p* < 0.05, Student's *t* test) altered protein levels. *B*, enrichment of protein domains according to the Pfam database within the 25 proteins having consensus sites for C-mannosylation and showing significantly altered secretion in DPY19L1 KO and/or DPY19L3 KO hiPSCs (*green bar*, Fisher exact test, *p* ≤ 0.05). *C*, relative protein levels of 11 thrombospondin type 1 domain–containing proteins identified in the secretome of hiPSCs (WT, DPY19L1 KO, and DPY19L3 KO). Dots represent fold changes of protein levels in individual KO samples compared with the mean WT protein levels. The mean of individual fold changes for each KO is depicted by bars (*blue bars* for DPY19L1KO and *red bars* for DPY19L3 KO). Statistically significant fold changes are indicated by *red asterisks*. hiPSCs, human induced pluripotent stem cells; TSR, thrombospondin type 1 repeat.

Numerous studies have shown that C-mannosylation supports secretion of target proteins. This effect was used in this study to identify novel C-mannosylated proteins that are potentially involved in early developmental processes by quantitative comparison of protein levels in cell culture supernatants of WT and derived DPY19L1- or DPY19L3-deficient hiPSCs. The secretomes of each of three replicates of WT hiPSCs as well as of the KO cell lines DPY19L1–C1, DPY19L1–C2, DPY19L3–C1, and DPY19L3–C2 were analyzed by LC–MS/MS and processed with the MaxQuant and Perseus software tools ((34, 35); supplemental Fig. S3). This led to the identification of 3078 distinct proteins in total. Upon filtering for extracellular proteins and defining more stringent parameters for identifications, we ended up with 398 proteins (supplemental Fig. S3*A*) of which the majority (342 proteins) were identified in all cell lines. A statistical analysis revealed that, compared with the WT, in the secretomes of DPY19L1 KO and DPY19L3 KO clones, the abundance of 33 and 52 proteins was significantly altered (*p* ≤ 0.05), respectively, whereas reduction of transcription by more than twofold was only observed for five of these proteins (supplemental Table S2). Screening of these proteins for the presence of the known C-mannosylation consensus sequences WxxW and/or WxxC led to the identification of 9 and 18 proteins significantly affected by the KO of DPY19L1 and DPY19L3, respectively, altogether 25 different proteins (Fig. 2*A*; supplemental Table S2 and supplemental Fig. S3*D*). Affirming our approach, one of these candidates, THBS1, is already known as a target protein for C-Man (41). As several proteins with TSRs are known from the literature to be C-mannosylated (10), we performed an enrichment analysis for protein domains of these 25 proteins (supplemental Table S2). This analysis revealed proteins containing TSP1 domains, which are synonymous to TSRs (42), to be significantly enriched in our candidate list (Fig. 2*B*). Based on this observation, we screened the entire list of 398 extracellular proteins for TSP1 domains in order to reveal more potential candidates that were not present in our initial screen. This analysis uncovered 11 proteins with TSP1 domains in total including several members of the ADAMTS family, of which several showed reduced abundance in the mutants' supernatants (Fig. 2*C*).

### THBS1 Is Endogenously C-mannosylated by hiPSCs

**Fig. 3:**
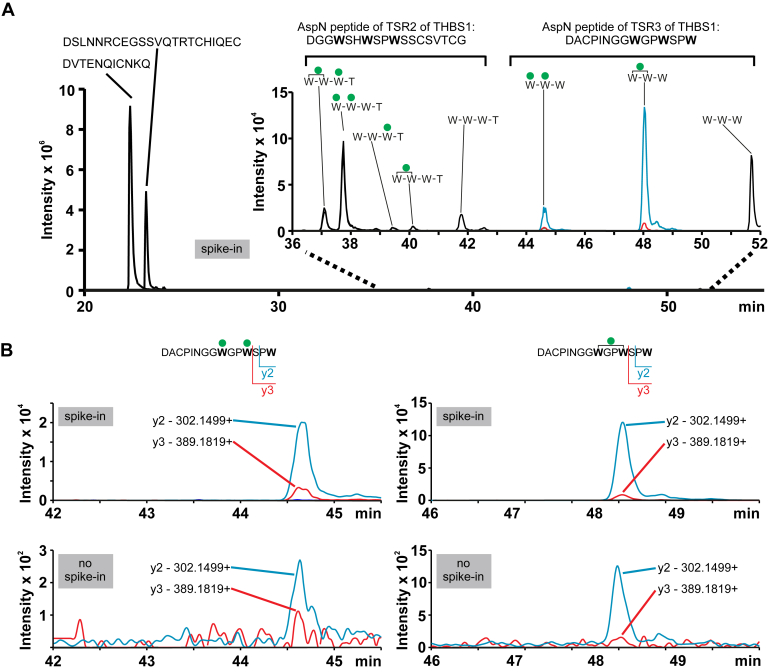
**Endogenous THBS1 is C-mannosylated by hiPSCs.***A*, chromatographic separation and MRM monitoring of proteolytic peptides derived from a THBS1 fragment containing TSR1 to 3 (as shown in supplemental Fig. S2*B*) recombinantly expressed in HEK 293T cells. Peptides containing the consensus site for C-mannosylation (WxxW/C) with no, one, or two mannoses, elute noticeably later than the two other TSR-derived peptides and are detected at significantly lower signal intensities. *B*, EICs of characteristic fragment ions derived from the mono-C-mannosylated (*right panels*) and di-C-mannosylated (*left panels*) TSR3 peptide from hiPS cell culture supernatants containing recombinant THBS1 (*upper panels*) as a positive control (spike-in) are compared with native samples of hiPS cell culture supernatants without addition of THBS1 (*lower panels*). In the native samples (no spike-in), the corresponding C-mannosylated TSR3 peptides were detected with low intensities at expected elution times, confirming the presence of endogenous C-mannosylated THBS1 in hiPSC cell culture supernatants. EICs were transformed in Skyline using the Savitzky–Golay smoothing. EICs, extracted ion chromatograms; HEK, human embryonic kidney; hiPSCs, human induced pluripotent stem cells; MRM, multiple reaction monitoring; THBS1, thrombospondin 1; TSR, thrombospondin type 1 repeat.

Because of technical restrictions, no C-mannosylated peptides could be identified in the secretomics screen by MS. Thus, we set up a more sensitive targeted MRM approach (43) for detection of C-mannosylated THBS1 peptides from complex mixtures as a proof of concept. We used a THBS1 fragment comprising TSR1 to 3, which was recombinantly expressed in HEK 293T cells and digested with AspN, to determine chromatographic RTs and optimize MS parameters. We detected two peptides from TSR2 and TSR3 containing the consensus site for C-mannosylation in their non-, mono-, and di-C-mannosylated state. These peptides—irrespective of C-mannosylation—were detected at less than 3% of the intensity of two other THBS-derived peptides, which do not contain a WxxW motif (Fig. 3*A*). With the knowledge of the specific chromatographic RTs and the intensity patterns of fragment ions for the two peptides containing the WxxW sites for C-mannosylation from the recombinant THBS1, we now analyzed a native sample of hiPSC-derived proteins from cell culture supernatants by MRM. Indeed we detected the most intense fragment ions for the peptide DACPINGG**W**GP**W**SP**W** with one or two C-mannoses at the expected elution times and with the expected intensity profiles (Fig. 3*B*).

### Targeting dpy19l3 in Medaka Revealed Defects in Optic Fissure Fusion

**Fig. 4:**
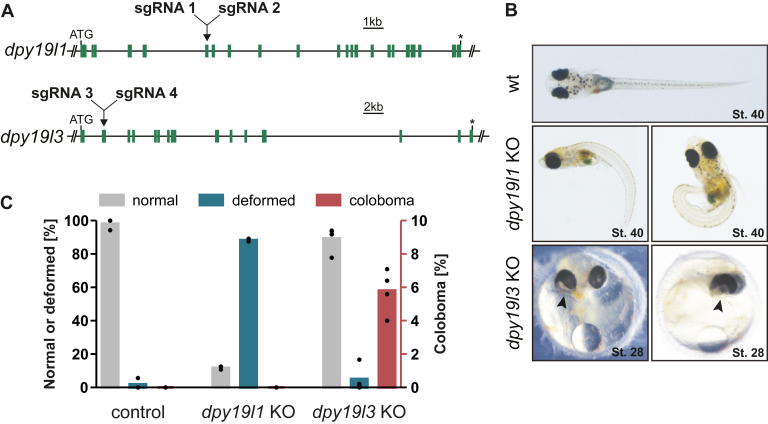
**Targeting of dpy19l3 in medaka caused coloboma.***A*, schemes of *dpy19l1* and *dpy19l3* genes indicating exons (*green bars*), sgRNA positions, stop codons (*asterisks*). *B*, micrographs of medaka WT as well as *dpy19l1* and *dpy19l3* deletion mutants stage 40 and stage 28 of embryonic development grown at 28 °C. The gross of dpy19l1 mutants show caudal bending and shortening of anterior–posterior axis. A proportion of *dpy19l3* mutants display defects in closure of the optic fissure, called coloboma (*arrows*). *C*, frequency of normal and deformed morphology as well as coloboma formation in medaka upon microinjection of GFP mRNA (control) or GFP mRNA in combination with sgRNAs targeting *dpy19l1* or *dpy19l3*, respectively. Bars indicating normal (*gray*) and deformed (*blue*) morphology refer to the left *y*-axis, bars for coloboma formation (*red*) refer to right *y*-axis. Results of individual injection experiments are represented by *dots*. sgRNA, single-guide RNA.

To assess the impact of defective C-mannosylation at an organismal level, we targeted the orthologs of human *DPY19L1* or *DPY19L3* in the medaka genome by zygotic microinjection of mRNA encoding Cas9 and two specific sgRNAs per gene at the one-cell stage (Fig. 4*A*). Targeting of *dpy19l1* caused major shortening of the anterior–posterior axis and caudal bending in more than 80% of the injected embryos (crispants; Fig. 4, *B* and *C*). In contrast, the vast majority of medaka embryos in which *dpy19l3* was targeted showed normal embryonic development. However, in about 5% of the *dpy19l3* crispants, the optic fissure did not close properly, a defect known as coloboma (Fig. 4, *B* and *C*). Interestingly, a similar coloboma phenotype was previously observed in zebrafish upon KO of ADAMTS16 (44). Since ADAMTS16 was also found to be less secreted in the secretome analysis of the C-mannosyltransferase–deficient hiPSCs (Fig. 2*C*), we hypothesized that ADAMTS16 is a target for C-mannosylation and that C-mannosylation of ADAMTS16 is important for its proper secretion, thereby affecting its function.

### Identification of ADAMTS16 as Target Protein for C-mannosylation

**Fig. 5:**
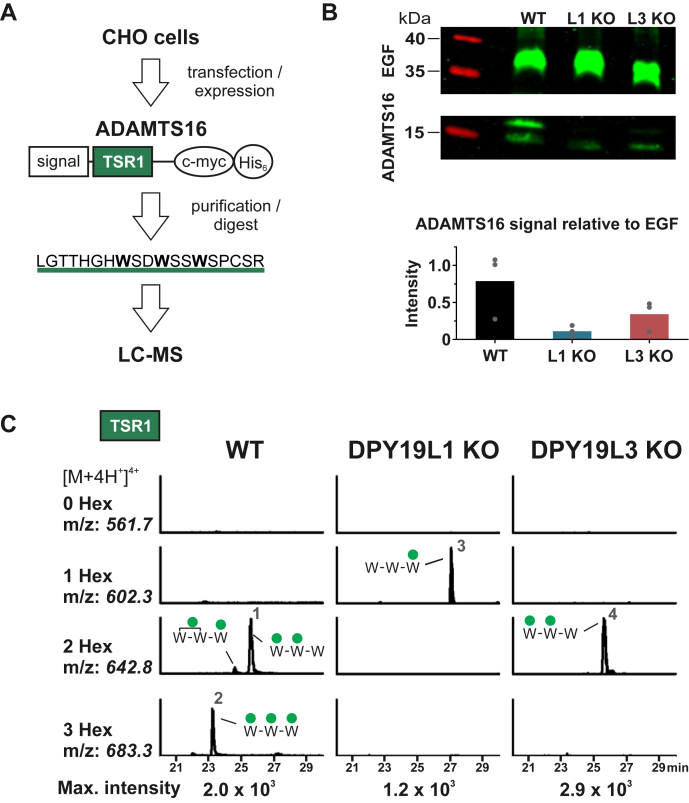
**ADAMTS16 is C-mannosylated by DPY19L1 and DPY19L3.***A*, scheme of the ADAMTS16 fragment expressed in WT, DPY19L1 KO, and DPY19L3 KO CHO-K1 cells. The fragment contains TSR1 of ADAMTS16 resulting in the indicated peptides upon tryptic digestion. *B*, Western blot of the TSR1-containing ADAMTS16 fragment secreted by WT and KO cells. Coexpression of a myc-tagged fragment of mouse Notch1 comprising EGF repeats 9–14 served as transfection and secretion control. The amount of secreted ADAMTS16 relative to secreted EGF is depicted in the bar graph; results of individual experiments are represented by *dots*. *C*, MS analysis of a tryptic digest of the purified ADAMTS16 fragment secreted from WT, DPY19L1 KO, or DPY19L3 KO CHO-K1 cells. Extracted ion chromatograms (EICs) of the TSR1-derived peptides ([M + H^+^]^4+^) with different numbers of hexoses (Hex) are displayed. Corresponding spectra of each sample were adjusted to the intensity of the most intense glycoform (maximum intensity, as indicated at the *bottom*). Annotation of peaks was based on the parental ion mass, RT, and fragmentation spectra. Numbers 1 to 4 refer to the fragmentation spectra of the respective peaks provided in supplemental Figure S5. For each peptide, only tryptophan (W) residues are depicted, with respective C-mannoses (*green circles*). ADAMTS, *A D*isintegrin *A*nd *M*etalloproteinase with *T*hrombo*S*pondin motifs; EGF, epidermal growth factor; RT, retention time; TSR, thrombospondin type 1 repeat.

In order to analyze C-mannosylation of ADAMTS16, its TSR1 (Fig. 5*A*) was recombinantly expressed in CHO-K1 WT, DPY19L1 KO, and DPY19L3 KO cells (7). Western blot analysis of the cell culture supernatant showed that the ADAMTS16 fragment is less secreted in the DPY19L1- and DPY19L3-deficient CHO-K1 cells (Fig. 5*B*). MS analysis of the secreted TSR1 of ADAMTS16 revealed that the **W**SD**W**SS**W**SPC motif of TSR1 of ADAMTS16 can be C-mannosylated at all three tryptophan residues. Deletion of DPY19L1 prevented C-mannosylation of the two first tryptophans, whereas C-mannosylation of the third tryptophan was not detected in the DPY19L3 mutant (Fig. 5*C*). Interestingly, the non–C-mannosylated TSR1 peptide of ADAMTS16 was not detected at all suggesting that it is not secreted without C-mannosylation.

## Discussion

More than 2500 proteins harboring C-mannosylation sites were predicted by bioinformatics from the human genome (8), but to date, only few proteins have actually been proven to be C-mannosylated. At the first glimpse, protein C-mannosylation seems to be well suited to be investigated by global proteomics screens, even from complex mixtures, as the modification does not dissociate during ionization or fragmentation processes and confers a defined mass of 162.05 Da to a tryptophan residue, which can be considered as a variable modification during database searches. We and others have already shown that C-mannosylated peptides of recombinantly expressed proteins can be detected by MS, and that this even enables proper assignment of the modified tryptophan residue upon fragmentation (6, 7, 17, 41, 45). Global MS-based screens have been widely applied for detection of other types of protein glycosylation such as O-mannosylation (46, 47) or O-GlcNAcylation (48, 49). However, glycoproteomic approaches to uncover the C-mannosylome of cells or tissues are scarce. Only very recently, an antibody against tryptophan C-mannosylation was described, which enabled the first proteomic assessment of a murine brain C-glycome upon enrichment of C-mannosylated peptides (13). In this study, we show that MS detects peptides with target sequences for C-mannosylation at much lower intensities than other peptides from the same protein. We attribute this to the high density of tryptophan residues causing an inherent hydrophobic character of peptides with WXXW motives, as it is widely accepted that hydrophobic peptides hardly ionize by ESI (50). Furthermore, we observe different numbers and distributions of C-mannose residues for individual peptides, which lower the detection levels of discrete peptide species. Thus, we argue that detection of C-mannosylated peptides is impeded by commonly used MS routines that depend on an intensity-based selection of precursor ions for fragmentation. Accordingly, for THBS1, which is a known target protein for C-mannosylation (41), and known to be secreted at high levels by human embryonic stem cells (51), we detected numerous peptides in our secretomics approach but none of the peptides with the consensus sites for C-mannosylation. It was necessary to establish a highly sensitive MRM assay to show the C-mannosylation of endogenous THBS1 from cell culture supernatants of hiPSCs, which is—to the best of our knowledge—the first direct evidence of C-mannosylation of an endogenous protein derived from cultured cells. However, MRM analysis cannot be applied for an unbiased large-scale screening to detect C-mannosylated peptides. Thus, to identify novel C-mannosylated proteins, we made use of the prevalent finding that protein secretion depends on C-mannosylation and quantitatively compared the secretomes of hiPSCs as well as derived C-mannosyltransferase deletion mutants.

The quantitative secretomics analysis revealed for the majority of significantly affected proteins a reduced secretion in the C-mannosyltransferase–deficient hiPSC models, and identification of THBS1 (41) can be considered as a positive control of our approach. However, secretion of some proteins, for example, ADAMTS8 was significantly augmented in the mutants that are probably caused by indirect effects of other affected proteins. We did not observe any obvious phenotype in the C-mannosyltransferase–deficient hiPSCs, but aberrant glycosylation often leads to defects in organismal development without affecting cultured cells (52, 53, 54). Indeed, upon targeting of *dpy19l1*, in medaka embryos, major malformations appeared, suggesting a broad impact of dpy19l1-mediated C-mannosylation during ontogeny. Interestingly, *dpy19l3* crispant medaka embryos partially developed ocular coloboma, that is a gap caused by incomplete closure of the optic fissure. A similar phenotype was recently shown in zebrafish upon morpholino-based KO of *adamts16* (44). Accordingly, we identified ADAMTS16 to be less secreted in the C-mannosyltransferase–deficient hiPSCs. ADAMTS16 belongs to the family of secreted metalloproteinases, which are involved in tissue and extracellular matrix remodeling (12), and was shown to be specifically expressed at the ventral optic cup prior to closure of the optic fissure during development of mice and zebrafish (44). Thus, ADAMTS16 was suggested to be involved in degradation of the basement membrane at the optic fissure edges, the prerequisite for optic fissure closure (44). ADAMTS16 was further shown to be an activator of the latent transforming growth factor-β signaling (55). Concerted regulation of the transforming growth factor-β signaling pathway is crucial for embryonic development, as for instance for the morphogenesis of the eye (56, 57). Considering the sequence homology of human and medaka ADAMTS16 (supplemental Fig. S2), these findings suggest that secretion of ADAMTS16 is reduced in the *dpy19l3* crispant medaka embryos. Consequently, its levels in the extracellular milieu of the developing eye may not be sufficient to fulfill its functions. The low prevalence of the coloboma phenotype in our medaka model can be explained as a result of both, the mosaic organism generated upon injection of the CRISPR/Cas9 system and the non–cell autonomous effect of the secreted ADAMTS16 protease. In this study, we show that TSR1 of ADAMTS16 is C-mannosylated by DPY19L1 and DPY19L3. We also obtained strong evidence that secretion of ADAMTS16 depends on C-mannosylation as the secretion levels of the recombinantly expressed ADAMTS16 TSR1 as well as the endogenous ADAMTS16 were both reduced in C-mannosyltransferase–deficient CHO-K1 and hiPS cells, respectively.

Taken together, we identified ADAMTS16 as a novel target protein for C-mannosylation and showed that C-mannosylation indirectly affects its function by affecting secretion of ADAMTS16.

## Data availability

All data are available in the main text or the supplemental files. The MRM data have been deposited to the ProteomeXchange Consortium *via* the Panorama Public partner repository (58) with the dataset identifier PXD024117. The MS proteomics data have been deposited to the ProteomeXchange Consortium *via* the PRIDE partner repository (59) with the dataset identifier PXD024192 for Q-TOF data and PXD024193 for Orbitrap data. Annotated fragmentation spectra resulting from Orbitrap analysis (best spectrum for any given peptide sequence) are available at the MS Viewer repository with the search key p5vtjou6lz.

## Supplemental data

This article contains supplemental data.

## Conflict of interest

The authors declare no competing interests.
