# Supplementary material for: The C-Mannosylome of Human Induced Pluripotent Stem Cells Implies a Role for ADAMTS16 C-Mannosylation in Eye Development
Source: Mol Cell Proteomics. 2021 May 8;20:100092. doi: 10.1016/j.mcpro.2021.100092 (PMC8256286; doi:10.1016/j.mcpro.2021.100092)
Supplement: Supplemental Figure S1 [file mmc1.pdf]

A

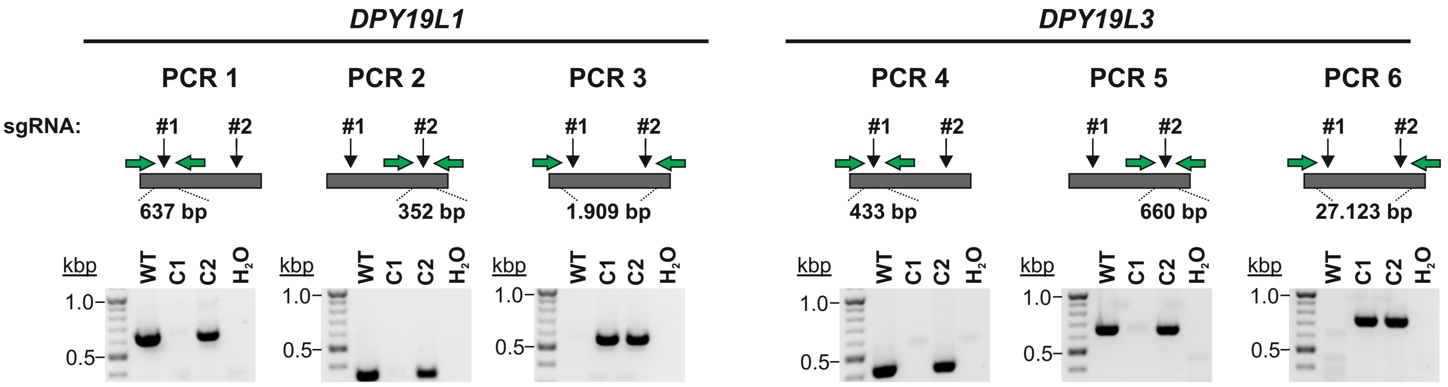

B

Primers used for screening of mutants

According to Figure S1

Gene	PCR #	Primer fw	Primer rv	PCR Product (WT)	Expected PCR Product (Macrodeletion)
DPY19L1	1	AGGCTATATTTGAGACTCCACTCA	GTAGATGAAGGAAAACTGGCTGTG	576 bp	-
	2	GTTCCTGTATCCCAAGTTTCCTGA	TCCTCTATGATATACCTCAGGGAG	352 bp	-
	3	AGGCTATATTTGAGACTCCACTCA	TCCTCTATGATATACCTCAGGGAG	1,909 bp	637 bp
DPY19L3	4	AATCCCAGCACTTTAGGATCCC	CCGGCACAGTTTAACAACCG	433 bp	-
	5	CTGTCACGGACTTCCTTCCC	AAGAGATGAGATATGCGGCTGG	660 bp	-
	6	AATCCCAGCACTTTAGGATCCC	AAGAGATGAGATATGCGGCTGG	27,123 bp	724 bp

C

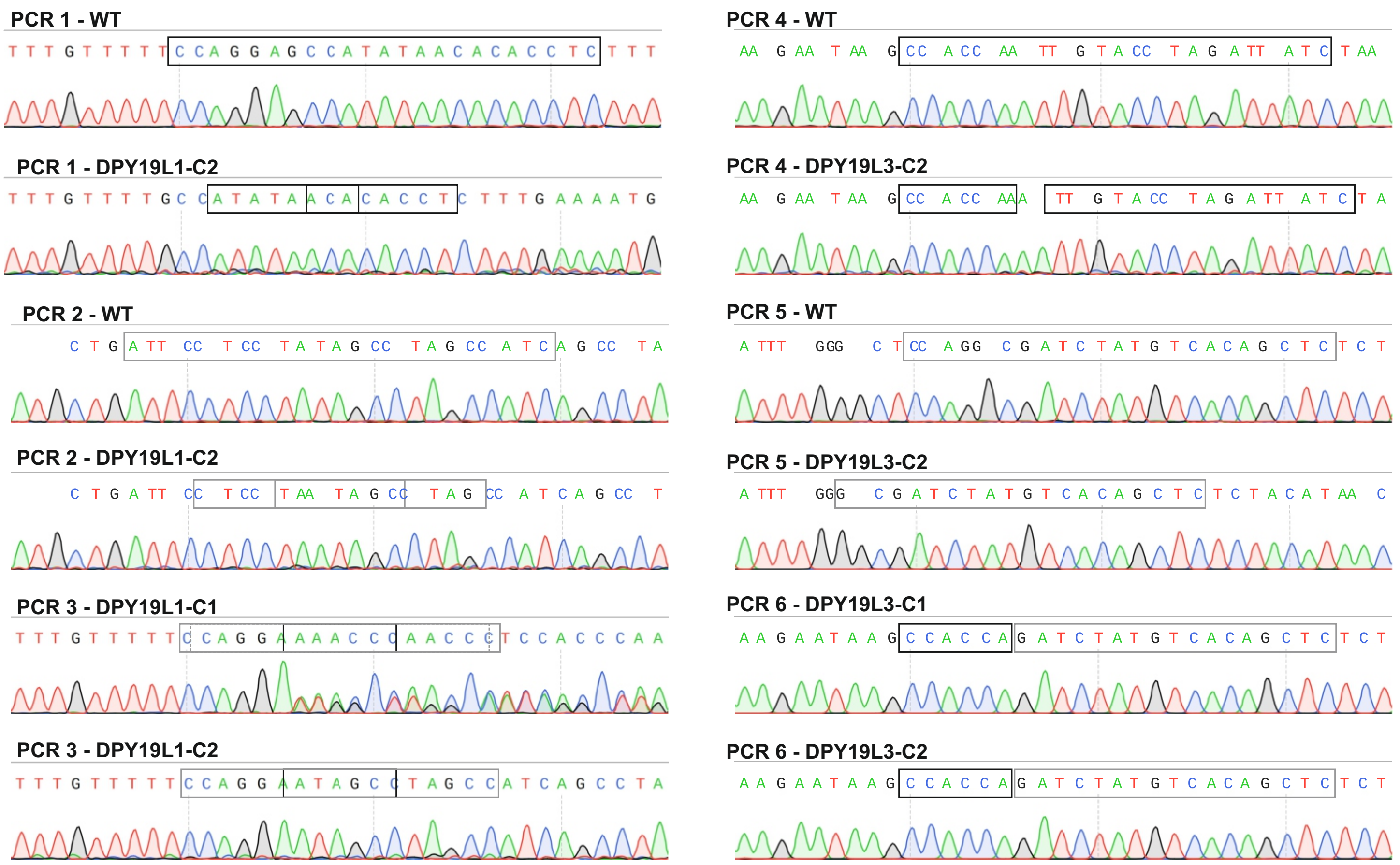

D

Expected protein sequences in WT, DPY19L1 KO and DPY19L3 KO clones

(Stars indicate stop codons. The section from sgRNA 1 to sgRNA 2 is boxed)

DPY19L1 WT

Alleles 1+2

MEGRPPPEGRPPPRPRTGRAPRGRRRAVFAAVLHWSHITHLFENDRHFSHLSTLEREMAFRTEMGLYYSYFKTIVEAPSFL
NGVWMIMNDKLTEYPLVINTLKRFNLYPEVILASWYRIYTKIMDLIGIQTKICWTVTRGEGLSPIESCEGLGDPACFYVAVI
FILNGLMMALFFIYGTYLSGSRLGGLVTVLCFFFNHGECTRVMWTPPLRESFSYPFLVLQMLLVTHILRATKLYRGSLIALC
ISNVFFMLPWQFAQFVLLTQIASLFAVYVVGYIDICKLRKIIYIHMISLALCFVLMFGNSMLLTSYYASSLVIIWGILAMKP
HFLKINVSELSLWVIQGCFWLFGTVILKYLTSKIFGIADDAHIGNLLTSKFFSYKDFDTLLYTCAAEFDFMEKETPLRYTKT
LLLPVVLVVFVAIVRKIISDMWGVLAKQQTHVRKHQFDHGELVYHALQLLAYTALGILIMRLKLFLTPHMCVMASLICSRQL
FGWLFCKVHPGAIVFAILAAMSIQGSANLQTQWNIVGEFSNLPQEELIEWIKYSTKPDAVFAGAMPTMASVKLSALRPIVNH
PHYEDAGLRARTKIVYSMYSRKAAEEVKRELIKLKVNYYILEESWCVRRSKPGCSMPEIWDVEDPANAGKTPLCNLLVKDSK
PHFTTVFQNSVYKVLEVVKE*

DPY19L1 KO – Clone 1

Alleles 1+2

MEGRPPPEGRPPPRPRTGRAPRGRRRAVFAAVLHWRTILFLFQDYCGSTLIFEWSMDDYE**TD*IPPCH*YIKKIQSLP*
GNFGQLVPDLYQNNGLDWYSNQDMLDGYQRRRTQSY*KL*RIGRSCLLLCCCNFYFKWTNDGIILHIWHIFKWQPIRRPGYS
VVLLFQSWRVYPCNVDTTSP*KLLISISCSSDVASDSYSQGYKTL*RKLDCTLHFQCIFHASLAVCSVCTSYSDCIIICSIC
CRVH*YM*ITEDHLYTHDFSCTLFCFDVWELNVINFLLCFFFGNYLGYSGNETTFPENKCI*T*FMGYSRMFLVIWNCHT*I
LDI*NFWYCR*RSYWQLTNIKIL*L*GF*YFIVYLCSGV*LYGKRDSTEIHKDIIASSCSCSVCCYC*KDY**YVGCLS*TT
DTCKKTPV*SWRAGLPCIAIVSIYSPWYFNYETKTLLDTTHVCYGITDLLKTAIWMALLQSTSWCYCVCYISSNVNTRFSKS
ANPVEYCRGVQQFAPRRTYRMDQI*Y*TRCSVCGCHAHDGKC*ALCTSAHCESSTL*RRRLESQNKNSILNV*SESSRRSEA
RTDKVKSELLHSRRVMVCKKIQAWLQYA*NLGCRRSCQCWENSLM*PLGEGFQTSLHHCIPEQCLQSPRSCKRM

DPY19L1 KO – Clone 2

Allele 1

MEGRPPPEGRPPPRPRTGRAPRGRRRAVFAAVLHWRTILFLFQDYCGSTLIFEWSMDDYE**TD*IPPCH*YIKKIQSLP*
GNFGQLVPDLYQNNGLDWYSNQDMLDGYQRRRTQSY*KL*RIGRSCLLLCCCNFYFKWTNDGIILHIWHIFKWQPIRRPGYS
VVLLFQSWRVYPCNVDTTSP*KLLISISCSSDVASDSYSQGYKTL*RKLDCTLHFQCIFHASLAVCSVCTSYSDCIIICSIC
CRVH*YM*ITEDHLYTHDFSCTLFCFDVWELNVINFLLCFFFGNYLGYSGNETTFPENKCI*T*FMGYSRMFLVIWNCHT*I
LDI*NFWYCR*RSYWQLTNIKIL*L*GF*YFIVYLCSGV*LYGKRDSTEIHKDIIASSCSCSVCCYC*KDY**YVGCLS*TT
DTCKKTPV*SWRAGLPCIAIVSIYSPWYFNYETKTLLDTTHVCYGITDLLKTAIWMALLQSTSWCYCVCYISSNVNTRFSKS
ANPVEYCRGVQQFAPRRTYRMDQI*Y*TRCSVCGCHAHDGKC*ALCTSAHCESSTL*RRRLESQNKNSILNV*SESSRRSEA
RTDKVKSELLHSRRVMVCKKIQAWLQYA*NLGCRRSCQCWENSLM*PLGEGFQTSLHHCIPEQCLQSPRSCKRM

Allele 2

MEGRPPPEGRPPPRPRTGRAPRGRRRAVFAAVLHWPYNTPL*K*PSFFSPLNIGKGDGFSH*NGTILFLFQDYCGSTLIFE
WSMDDYE**TD*IPPCH*YIKKIQSLP*GNFGQLVPDLYQNNGLDWYSNQDMLDGYQRRRTQSY*KL*RIGRSCLLLCCCNF
YFKWTNDGIILHIWHIFKWQPIRRPGYSVVLLFQSWRVYPCNVDTTSP*KLLISISCSSDVASDSYSQGYKTL*RKLDCTLH
FQCIFHASLAVCSVCTSYSDCIIICSICCRVH*YM*ITEDHLYTHDFSCTLFCFDVWELNVINFLLCFFFGNYLGYSGNETT
FPENKCI*T*FMGYSRMFLVIWNCHT*ILDI*NFWYCR*RSYWQLTNIKIL*L*GF*YFIVYLCSGV*LYGKRDSTEIHKDI
IASSCSCSVCCYC*KDY**YVGCLS*TTDTCKKTPV*SWRAGLPCIAIVSIYSPWYFNYETKTLLDTTHVCYGITDLLKTAI
WMALLQSTSWCYCVCYISSNVNTRFSKSANPVEYCRGVQQFAPRRTYRMDQI*Y*TRCSVCGCHAHDGKC*ALCTSAHCESS
TL*RRRLESQNKNSILNV*SESSRRSEARTDKVKSELLHSRRVMVCKKIQAWLQYA*NLGCRRSCQCWENSLM*PLGEGFQT
SLHHCIPEQCLQSPRSCKRM

DPY19L3 WT

Alleles 1+2

MMSIRQRREIRATEVSEDFPAQEENVKLENKLPSGCTSRRLWKILSLTIGGTIALCIGLLTSVYLATLHENDLWFSNIKEV
EREISFRTECGLYYSYYKQMLQAPTLVQGFHGLIYDNKTESMKTINLLQRMNIYQEVFLSILYRVLPIQKYLEPVYFYIYT
LFGLQAIYVTALYITSWLLSGTWLSGLLAAFWYVTNRIDTTRVEFTIPLRENWALPFFAIQIAAITYFLRPNLQPLSERLT
LLAIFISTFLFSLTWQFNQFMMLMQALVLFTLDSLDMLPAVKATWLYGIQITSLLLVCILQFFNSMILGSLLISFNLSVFIA
RKLQKNLKTGSFLNRLGKLLLHLFMVLCLTLFLNNIIKKILNLKSDEHIFKFLKAKFGLGATRDFDANLYLCEEAFGLLPFN
TFGRLSDTLLFYAYIFVLSITVIVAFVVAFHNLSDSTNQQSVGKMEKGTVDLKPETAYNLIHTILFGFLALSTMRMKYLWTS
HMCVFASFGLCSPEIWELLLKSVHLYNPKRICIMRYSVPILILLYLCYKFWPGMMDELSELREFYDPDTVELMNWINSNTPR
KAVFAGSMQLLAGVKLCTGRTLTNHPHYEDSSLRERTRAVYQIYAKRAPEEVHALLRSFGTDYVILEDSICYERRHRRGCRL
RDLLDIANGHMMDGPGENDPDLKPADHPRFCEEIKRNLPPYVAYFTRVFQNKTFHVYKLSRNK*

DPY19L3 KO – Clone 1

Alleles 1+2

MMSIRQRREIRATEVSEDFPAQEENVKLENKLPSGSMSQLST*PAGYSVVHGCQDCWQLSGMSQIE*IPQELSLPSH*GRT
GRCHSLQFR*QQLHIS*DQTYSLFLKG*HFLPFSYQLFSLV*HGNLINL*C*CKH*CCSHWTPWTCCQQ*RRHGCMEYR*QV
YSWSAFFSFLIP*FLDHCLSVLTFQYSLQENFRKI*KLEASLIGLGNFCYIYLWFYV*HFFSTT*LRKFLT*SQMNTYLNF*
RQNLGLEQQGILMQISICVKKLLASCLLIHLEGFQILCFFMLTYSFCPSQ*L*HSLLPFIISVILQINNPWVKWKKAQLT*N
QKLPTT*YIPFCLDSWH*VQ*E*STSGRHTCVCSHHSAYVALKYGSYF*SQSIFITQRGYV*CDIQYRY*YCCIYAISSGQE
*WMNSPS*ENSMIQIQWS**TGLTLTLQERLCLREACSCWPESSCAREGP*PTTRTMKTAA*ESGPERFIRYMPRGHQRKCM
PS*GPSALTT*SWKTASATSGGTAGAADSGTCWTLPTAT*WMAQERMILI*NLQTTLASVKRSKETCLPTWPTSPECSRTKP
STFTSCPETS

DPY19L3 KO – Clone 2

Allele 1

MMSIRQRREIRATEVSEDFPAQEENVKLENKLPSGSMSQLST*PAGYSVVHGCQDCWQLSGMSQIE*IPQELSLPSH*GRT
GRCHSLQFR*QQLHIS*DQTYSLFLKG*HFLPFSYQLFSLV*HGNLINL*C*CKH*CCSHWTPWTCCQQ*RRHGCMEYR*QV
YSWSAFFSFLIP*FLDHCLSVLTFQYSLQENFRKI*KLEASLIGLGNFCYIYLWFYV*HFFSTT*LRKFLT*SQMNTYLNF*
RQNLGLEQQGILMQISICVKKLLASCLLIHLEGFQILCFFMLTYSFCPSQ*L*HSLLPFIISVILQINNPWVKWKKAQLT*N
QKLPTT*YIPFCLDSWH*VQ*E*STSGRHTCVCSHHSAYVALKYGSYF*SQSIFITQRGYV*CDIQYRY*YCCIYAISSGQE
*WMNSPS*ENSMIQIQWS**TGLTLTLQERLCLREACSCWPESSCAREGP*PTTRTMKTAA*ESGPERFIRYMPRGHQRKCM
PS*GPSALTT*SWKTASATSGGTAGAADSGTCWTLPTAT*WMAQERMILI*NLQTTLASVKRSKETCLPTWPTSPECSRTKP
STFTSCPETS

Allele 2

MMSIRQRREIRATEVSEDFPAQEENVKLENKLPSGCTSRRLWKILSLTIGGTIALCIGLLTSVYLATLHENDLWFSNIKEV
EREISFRTECGLYYSYYKQMLQAPTLVQGFHGLIYDNKTESMKTINLLQRMNIYQEVFLSILYRVLPIQKYLEPVYFYIYT
LFGRSMSQLST*PAGYSVVHGCQDCWQLSGMSQIE*IPQELSLPSH*GRTGRCHSLQFR*QQLHIS*DQTYSLFLKG*HFL
PFSYQLFSLV*HGNLINL*C*CKH*CCSHWTPWTCCQQ*RRHGCMEYR*QVYSWSAFFSFLIP*FLDHCLSVLTFQYSLQEN
FRKI*KLEASLIGLGNFCYIYLWFYV*HFFSTT*LRKFLT*SQMNTYLNF*RQNLGLEQQGILMQISICVKKLLASCLLIHL
EGFQILCFFMLTYSFCPSQ*L*HSLLPFIISVILQINNPWVKWKKAQLT*NQKLPTT*YIPFCLDSWH*VQ*E*STSGRHTC
VCSHHSAYVALKYGSYF*SQSIFITQRGYV*CDIQYRY*YCCIYAISSGQE*WMNSPS*ENSMIQIQWS**TGLTLTLQERL
CLREACSCWPESSCAREGP*PTTRTMKTAA*ESGPERFIRYMPRGHQRKCMPS*GPSALTT*SWKTASATSGGTAGAADSGT
CWTLPTAT*WMAQERMILI*NLQTTLASVKRSKETCLPTWPTSPECSRTKPSTFTSCPETS

E

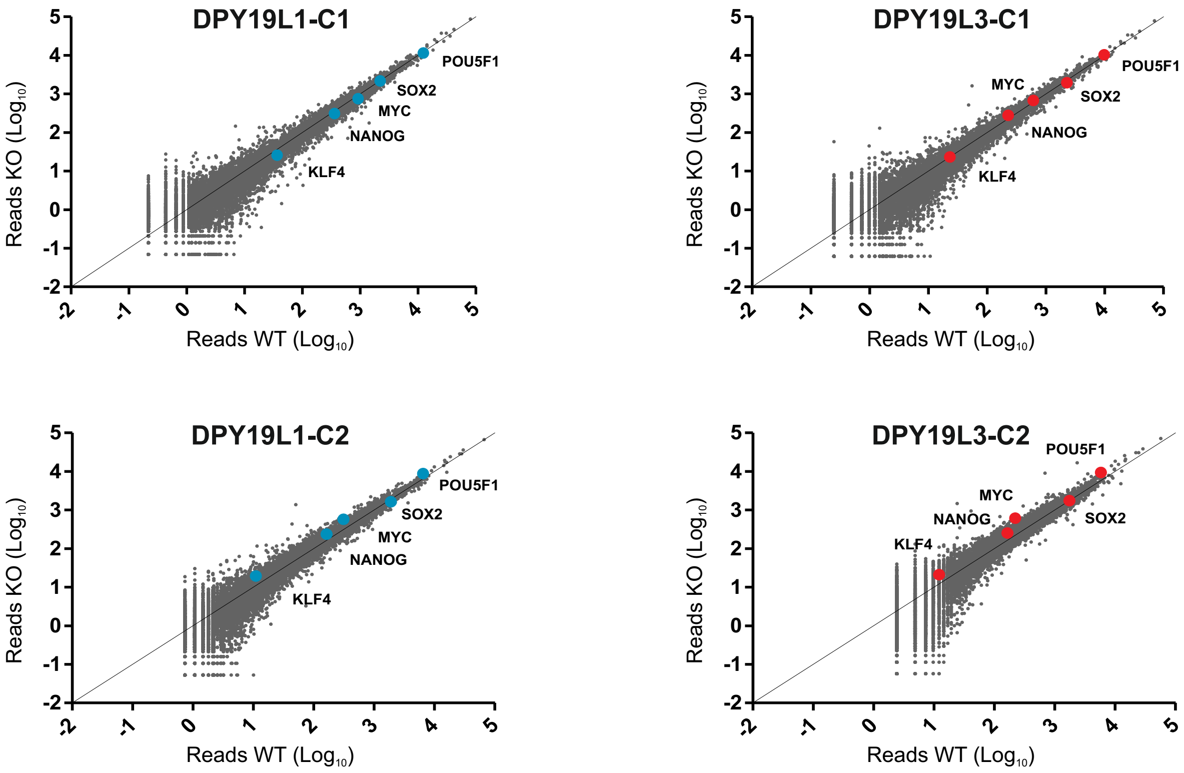

DPY19L1-C1
DPY19L3-C1
DPY19L1-C2
DPY19L3-C2
POU5F1
SOX2
MYC
NANOG
KLF4
Reads KO (Log10)
Reads WT (Log10)
