# Supplementary material for: The C-Mannosylome of Human Induced Pluripotent Stem Cells Implies a Role for ADAMTS16 C-Mannosylation in Eye Development
Source: Mol Cell Proteomics. 2021 May 8;20:100092. doi: 10.1016/j.mcpro.2021.100092 (PMC8256286; doi:10.1016/j.mcpro.2021.100092)
Supplement: Supplemental Figure S2 [file mmc2.pdf]

A Constructs for recombinant protein expression

pCAG-SecTagB

(PCR amplified section from pSecTagB to replace the eGFP in pCAG eGFP. Endonuclease BsmBI (underlined) cleaves DNA within the XhoI and BgIII (bold letters) restriction sites. KpnI and Not sites are boxed.)

ACGTCTC**CTCGAG**ACCATGGAGACAGACACACTCCTGCTATGGGTACTGCTGCTCTGGGTTCCAGGTTCCACTGGTGACGCG
GCCCAGCCGGCCAGGCGCGCGCCGTACGAAGCTTGGTACCGAGCTCGGATCCACTAGTCCAGTGTGGTGGAATTCTGCA
GATATCCAGCACAGTGGCGGCCGCTCGAGTCTAGAGGGCCCGAACAAAAACTCATCTCAGAAGAGGATCTGAATAGCGCCG
TCGACCATCATCATCATCATCATTGAGTTTAAACCCGCTGATCAGC**AGATCT**GAGACGC

pCAG-SecTag-hTHBS1-TSR_2_3

(N-terminal signal sequence, C-terminal c-myc epitope and His6 tag are underlined. The protein sequence of THBS1 cloned between KpnI and NotI sites of pCAG-SecTagB is boxed.)

METDTLLLWVLLLWVPGSTGDAAQPARRARRTKLGTFKQDGGWSHWSPWSSCSVTCGDGVITRIRLCNSPSPQMNGKPCEG
EARETKACKKDACPINGGWGPWSPWDICSVTCGGGVQKRSRLCNNPTPQFGGKDCVGDVTENQICNKQDCPISRPLESRGP
EQKLISEEDLNSAVDHHHHHH

pCAG-SecTag-ADAMTS16-TSR_1

(N-terminal signal sequence, C-terminal c-myc epitope and His6 tag are underlined. The protein sequence of ADAMTS16 cloned between KpnI and NotI sites of pCAG-SecTagB is boxed.)

METDTLLLWVLLLWVPGSTGDAAQPARRARRTKLGTTHGHWSDWSSWSPCSRTCGGGVSHRSRLCTNPKPSHGGKFCEGST
RTLKLCNSQKCPRDKRPLESRGPEQKLISEEDLNSAVDHHHHHH

pCMV-SecTag-hTHBS1-TSR_1_2_3

(N-terminal signal sequence, C-terminal c-myc epitope and His6 tag are underlined. The protein sequence of THBS1 cloned between HindIII and XbaI sites of pSecTagB is boxed.)

METDTLLLWVLLLWVPGSTGDAAQPARRARRTKLSADDGWSPWSEWTSCSTSCGNGIQQRGRSCDSLNNRCEGSSVQTRTC
HIQECDKRFKQDGGWSHWSPWSSCSVTCGDGVITRIRLCNSPSPQMNGKPCEGEARETKACKKDACPINGGWGPWSPWDIC
SVTCGGGVQKRSRLCNNPTPQFGGKDCVGDVTENQICNKQDCPISRGPEQKLISEEDLNSAVDHHHHHH

mouse Notch EGF repeat 9 to 14

(N-terminal signal sequence, C-terminal c-myc epitope and His6 tag are underlined. The protein sequence of NOTCH1 cloned between HindIII and XbaI sites of pSecTagB is boxed.)

METDTLLLWVLLLWVPGSTGDAAQPARRARRTKLNIDDCASAACFQGATCHDRVASFYCECPHGRTGLLCHLNDACISNPC
NEGSNCDTNPVNGKAICTCPSGYTGPACSQDVDECALGANPCEHAGKCLNTLGSFECQCLQGYTGPRCEIDVNECISNPCQ
NDATCLDQIGEFQCICMPGYEGVYCEINTDECASSPCLHNGHCMDKINEFQCQCPKGFNGHLCQYDVDECASTPCKNGAKC
LDGPNTYTCVCTEGYTGTHCEVDSRGPEQKLISEEDLNSAVDHHHHHH

B Alignment of ADAMTS16

Homo sapiens

Oryzias latipes

```
MK----PRARGWRGLAAL------WMLLAQVAEQAPACAMGPA-----------------
MSGTRVPCA-GWKRWTRLCVFMICWFCLCAPSRVGSLRAFTQSRLTTSFKNFLLLFQSAL
*.    * * **:  : *      *: *.  :. ..  *:  :

--------AAAPGSPSVPRPPPPAERPGWMEKGEYDLVSAYEVDHRGDYVSHEIMHHQRR
LVNTNEYRGASTLLQSLNLPP----------HTEYEIVAPYEVSHEGVYISHRVSHHERR
        .*:.   *:  **          : **::*:.***.*.* *:**.: **:**

RRAVPVS----------EVESLHLRLKGSRHDFHMDL-RTSSSLVAPGFIVQTLGKTGTK
RRRRSLTTEAQSSNKGGSSEKVHFRLSGLGQDFHMELWEASESLIAPGFTIQVLGKNSTK
**  .::          . *.:*:**.*  :****:* .:*.**:****  :*.***..**

SVQTLPPEDFCFYQGSLRSHRNSSVALSTCQGLSGMIRTEEADYFLRPLPSHLSWKLGRA
SLRAYHQDDLCFYQGSLRSRVNSSVALSTCMGMSGLIRTQHADYFLRPVSRSLAERENFT
*:::   :*:*********: ********* *:**:***:.*******:.  *: : . :

AQGSSPSHVLYKRST---------EPHA-------------PGASEVLVTSRTWELAHQP
APVTHQPHILYKRDGVTWLKQMYHQPRALQKRSADSFQHLSPRPSKV---NSPYDGAHQK
*  :  .*:****.          :*:*             *  .*:*    . .:: ***

--------LHS----------------------SDLRLGLPQKQHFCGRRKKYMPQPPKE
RVQRQQSGIHSHNSSNSSSLLDRRKQHGDVHHLSDYRPGEKQRQHFCGRKKKYMPKPPEE
        :**      ** * *  *:******:*****:**:*

DLFILPDEYKSCLRHKRSLLRSHRN-EELNVETLVVVDKKMMQNHGHENITTYVLTILNM
NIYVLPDEYKFIPRNKRAVLSKNRDIQRLNVETLVVVDRKMMDNHGHDNITTYALTVLNM
::::******   *:**::* .:*: :.**********:***:****:*****.**:***

VSALFKDGTIGGNINIAIVGLILLEDEQPGLVISHHADHTLSSFCQWQSGLMGKDGTRHD
VSSLFKDGTIGGNINIVIVGLILLDEEQDGLMINHHADHTLNSFCQWQSTLGGREGRHHD
**:*************.*******::** **:*.*******.******* * *::* :**

HAILLTGLDICSWKNEPCDTLGFAPISGMCSKYRSCTINEDTGLGLAFTIAHESGHNFGM
HAILLTGLDICSWKNEPCDTLGFAPISGMCSKYRSCTINEDTGLGLAFTIAHESGHNFGM
************************************************************

IHDGEGNMCKKSEGNIMSPTLAGRNGVFSWSPCSRQYLHKFLSTAQAICLADQPKPVKEY
VHDGEGNVCKKSEGNIMSPTLAGHNGIFSWSPCSRQYLSRFLNTAQALCLSDEPKAAKEY
:******:***************:**:*********** :**.****:**:*:**..***

KYPEKLPGELYDANTQCKWQFGEKAKLCMLDFKKDICKALWCHRIGRKCETKFMPAAEGT
RYPEKLPGELYDADTQCKWQFGEKAKLCTLDFKKDICKALWCHRVGRKCETKFMPAAEGS
:************:************** ***************:**************:
```

```
ICGHDMWCRGGQCVKYGDEGPKP THGHWSDWSSWSPCSRTCGGGVSHRSRLCTNPKPSHG
ACGPEMWCRRGQCVKQGDEGPGP QHGHWSEWSSWSACSRSCESGVTSRERQCSNPRPAYG
 ** :**** ***** ***** *  *****:*****.***:* .**: *.* *:**:*::*

GKFCEGSTRTLKLCNSQKCPRD SVDFRAAQCAEHNSRRFRGRHYKWKPYTQVEDQDLCKL
 GKFCEGSAKSYKLCNAVDCPPN STDYRAHQCAEFNSKQFRGWYYTWRPYTKVDDQDVCKL
*******::: ****: .** :*.*:** ****.**::*** :*.*:***:*:***:***

YCIAEGFDFFFSLSNKVKDGTPCSEDSRNVCIDGICERVGCDNVLGSDAVEDVCGVCNGN
YCFAEGYDFFFALASKVKDGTLCSKDNTNVCVDGLCERVGCDSVLGSTAVLDACGVCKGD
**:***:****:*:.****** **:*. ***:**:*******.**** ** *.****:*:

NSACTIHRGLYTKHHHTNQYYHMVTIPSGARSIRIYEMNVSTSYISVRNALRRYYLNGHW
NSTCKIYKGQYTKQHFTNQYYGVVTIPAGARSIRVMELNTSSSYLAVRDNHRHYYLNGHW
**:*.*::* ***:*.***** :****:******: *:*.*:**::**:   *:*******

TVDWPGRYKFSGTTFDYRRSYNEPENLIATGPTNETLIVELLFQGRNPGVAWEYSMPRLG
TVDWSGRHSIAGSIFEYKRPYNRPESLLSAGPTNESLIIEILLQGWNPGVRWEYTLSNTD
****.**:.::*: *:*:*.**.**.*:::*****:**:*:*:** **** ***::.. .

TE-KQPPAQPSYTWAIVRSECSVSCGGGQMTVREGCYRDLKFQVNMSFCNPKTRPVTGLV
DRLMKNPSKHNYSWAVVQSLCTVTCAGGRMSTNASCYKDMRVQVNTSYCNPKMKPATGVM
 .  : *:: .*:**:*:* *:*:*.**:*:.. .**:*::.*** *:**** :*.**::

PCKVSACPPSWSVGNWSACSRTCGGGAQSRPVQCTRRVHYDSEPVPA-SLCPQPAPSSRQ
PCNTQPCPHSWSVGEWGVCSRSCGGGEQTRQVQCVQRTNQNNVDARADSGCAQPAPVRRQ
**:...** *****:*..***:**** *:* ***.:*.: :.   . * * *.****  **

ACNSQSCPPAWSAGPWAECSHTCGKGWRKRAVACKSTNPSARAQLLPDAVCTSEPKPRMH
TCNTHSCPPVWSTGPWSQCSHKCGNGLKKRTVLCVSPKADAQTRTLPDSKCAGLQKPPSQ
:**::****.**:***::***.**:* :**:* * *.:..*::: ***: *:.  **  :

EACLLQRCHKPKKLQWLVSAWSQCSVTCERGTQKRFLKCAEKYVSGKYRELASKKCSHLP
EPCFIKRCQKQRKVQWFVSTWQECSAKCGQGYQARFIKCAEKDTAGKYRELPAKKCHHVP
*.*:::**:* :*:**:**:*.:**..* :* * **:***** .:******.:*** *:*

KPSLELERAC------------------------APLPCPRHPPFAAAGPSRGSWFASPWS
KPTVDLQRPCIVAECRSHTTPAVHQWVTPLRTYPPQPLPHSPP---------EWQSSPWS
**:::*:*.*       .* * *: **         .* :****

QCTASCGGGVQTRSVQCLAGGRPASGCLLHQKPSASLACNTHFCPIAEKKDAFCKDYFHW
HCTVTCGGGVQSRTVYCQVQGKLSSGCAPHLKPPMSQACNTNFCPQPEKKDLLCRDYFNW
:**.:******:*:* *  . *: :***   * **. * ****:*** .**** :*:***:*

CYLVPQHGMCSHKFYGKQCCKTCSKSNL
CYLVPQHGVCNHKFYGKQCCQSCSNSNL
********:*.*********::**:***
```
