# Supplementary figures and images for: The C-Mannosylome of Human Induced Pluripotent Stem Cells Implies a Role for ADAMTS16 C-Mannosylation in Eye Development
Source: Mol Cell Proteomics. 2021 May 8;20:100092. doi: 10.1016/j.mcpro.2021.100092 (PMC8256286; doi:10.1016/j.mcpro.2021.100092)

**A**

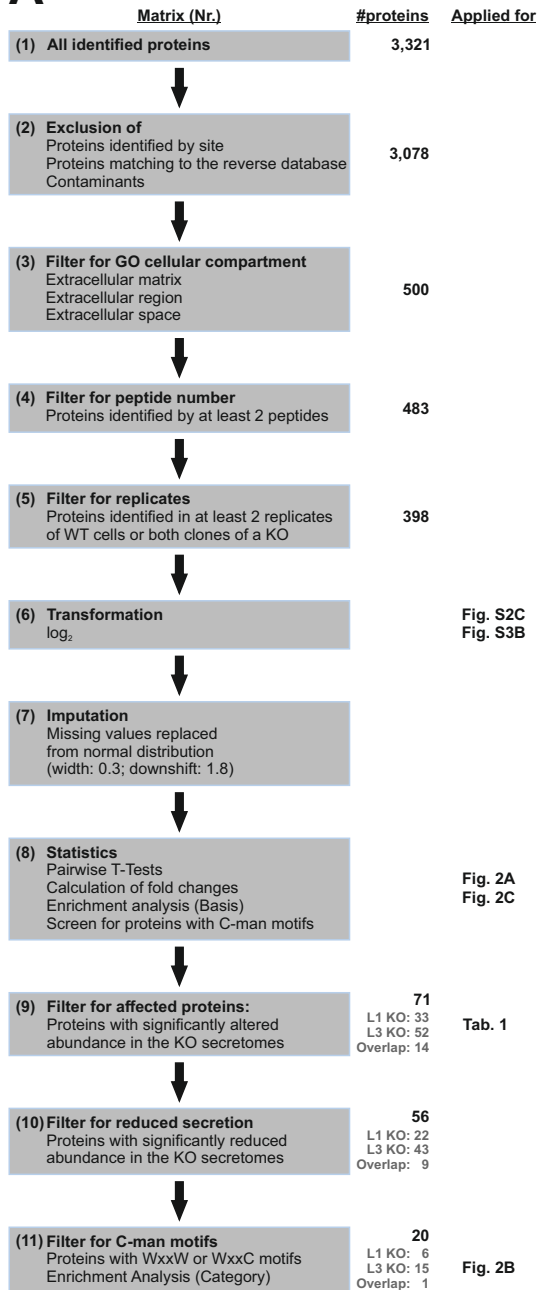

**B**

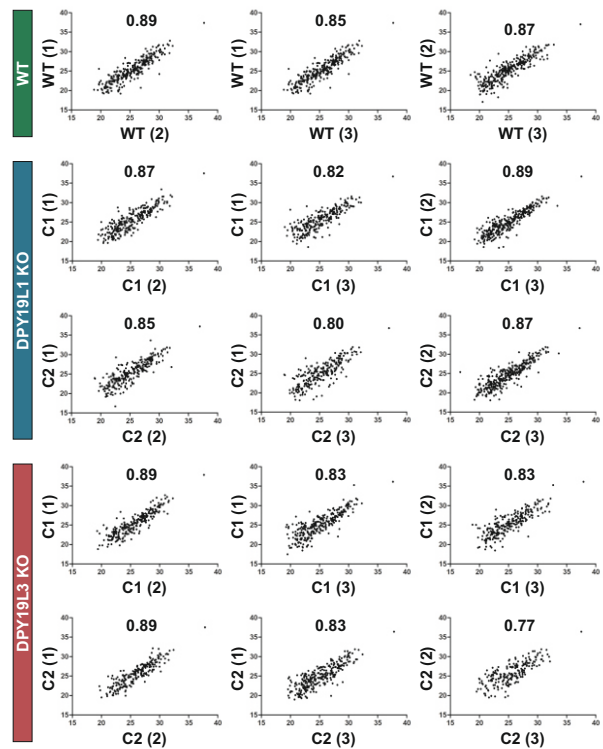

C

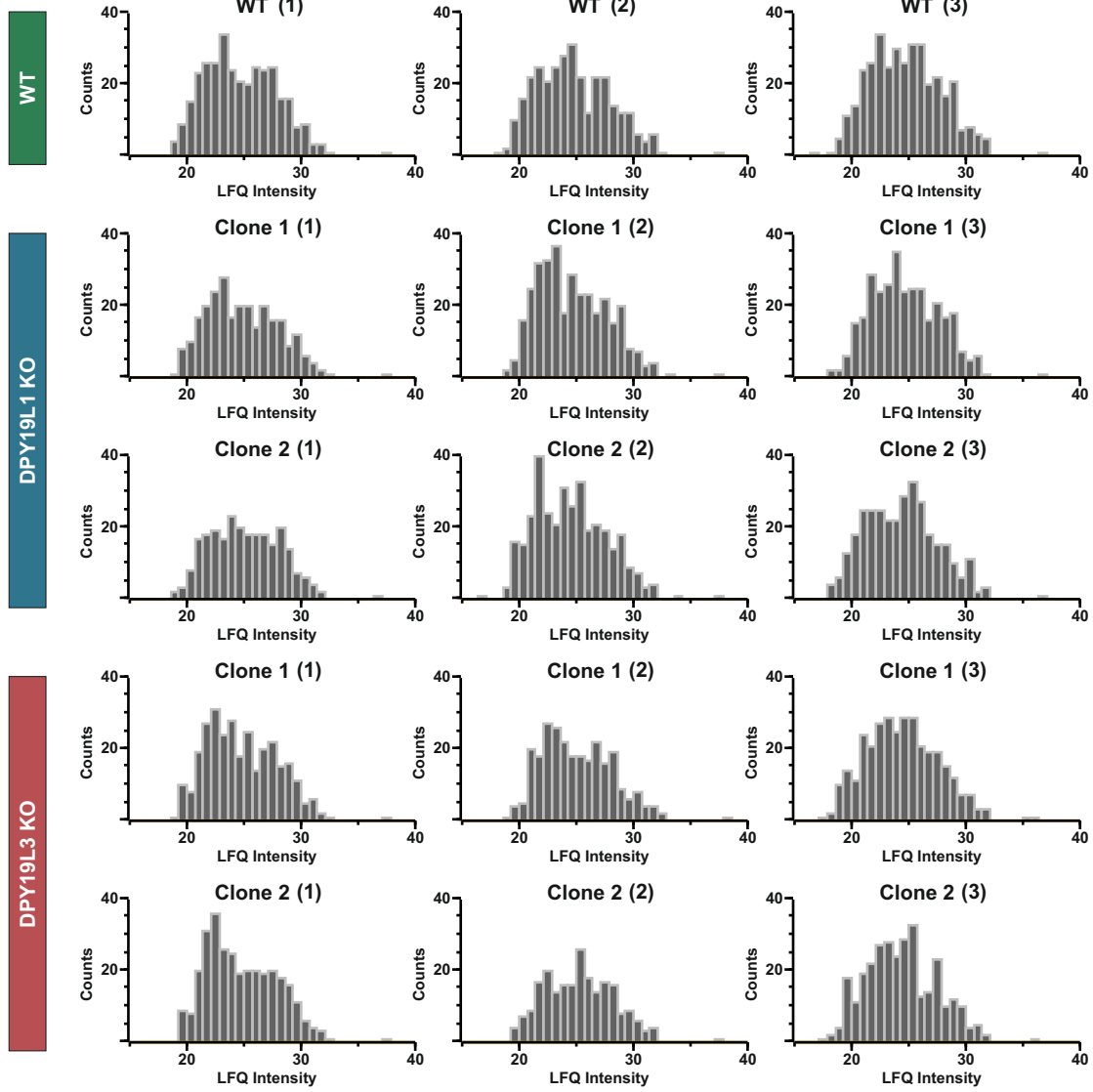

D

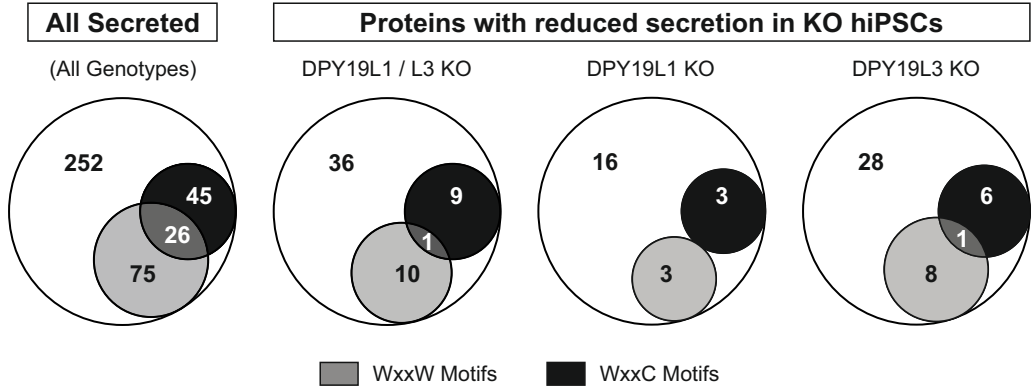

Supplement: Supplemental Figure S3 [file mmc3.pdf]

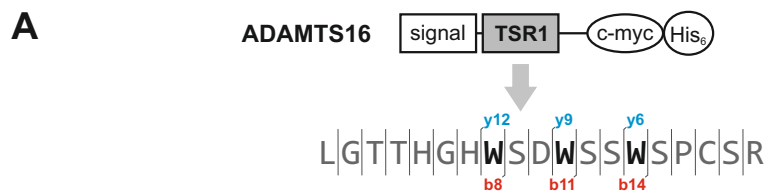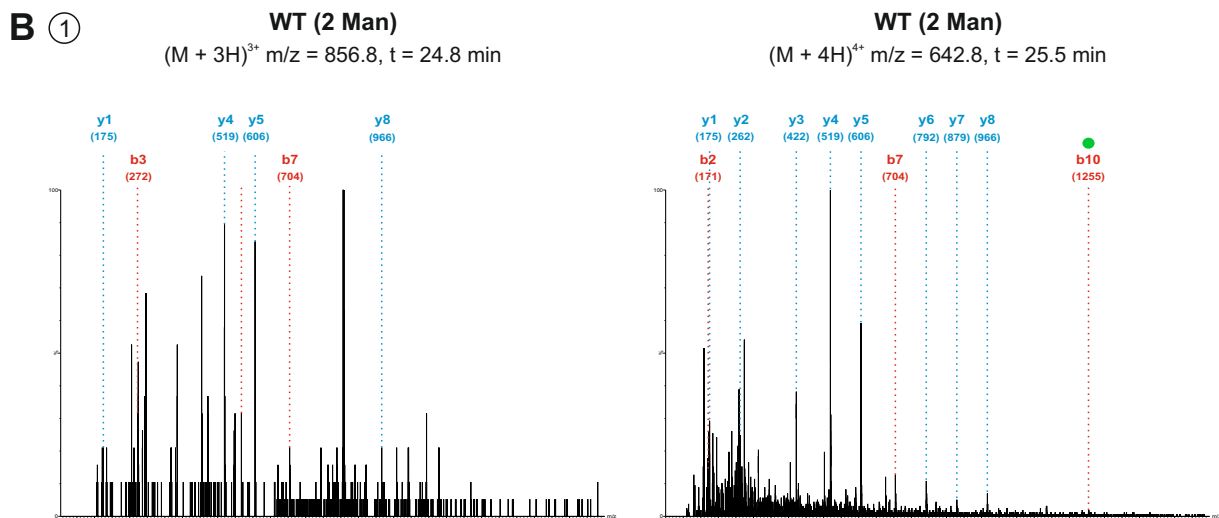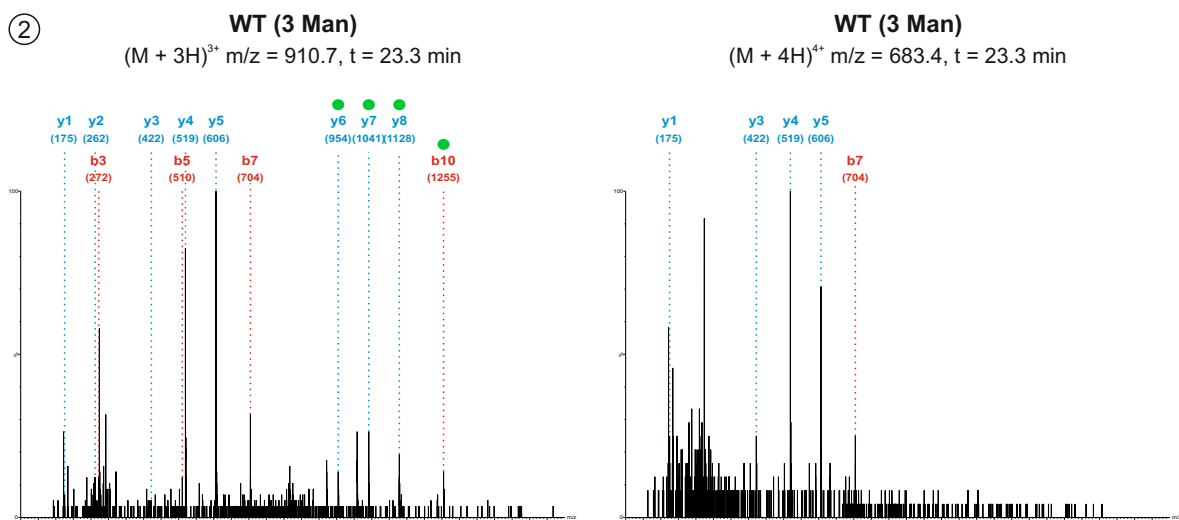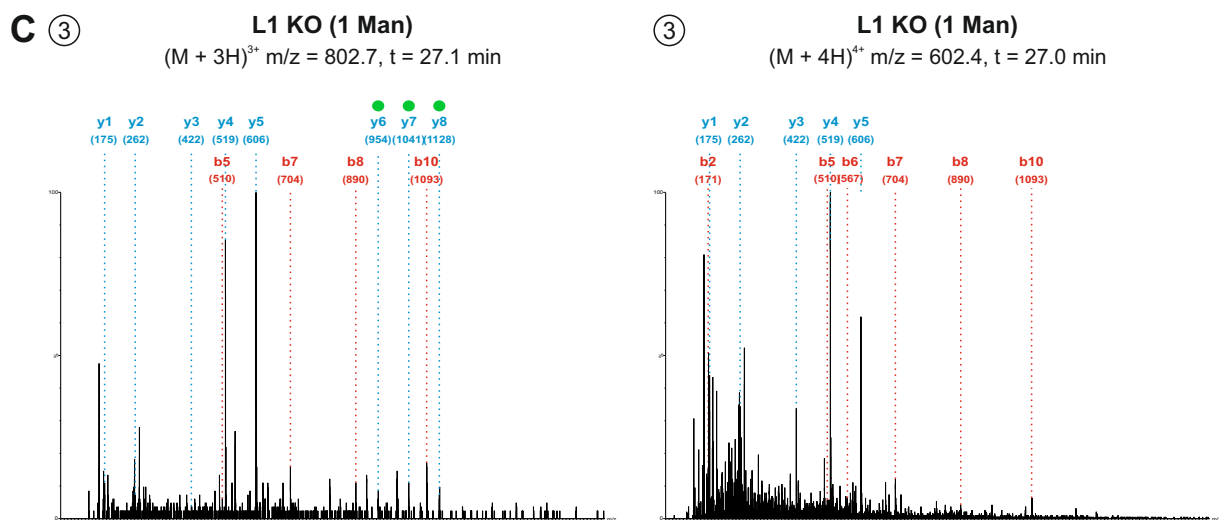

D ④

L3 KO (2 Man)

(M + 3H)<sup>3+</sup> m/z = 856.7, t = 25.7 min

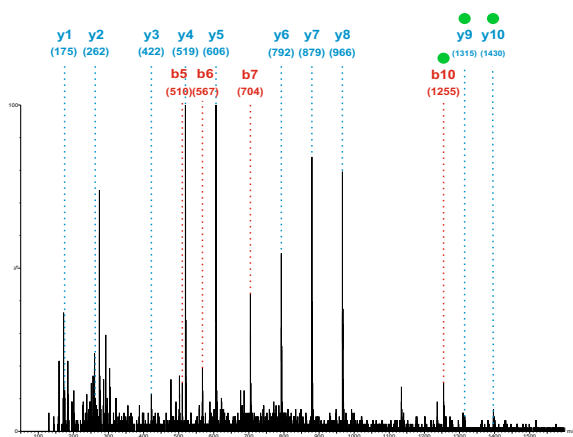

L3 KO (2 Man)

(M + 4H)<sup>4+</sup> m/z = 642.9, t = 25.6 min

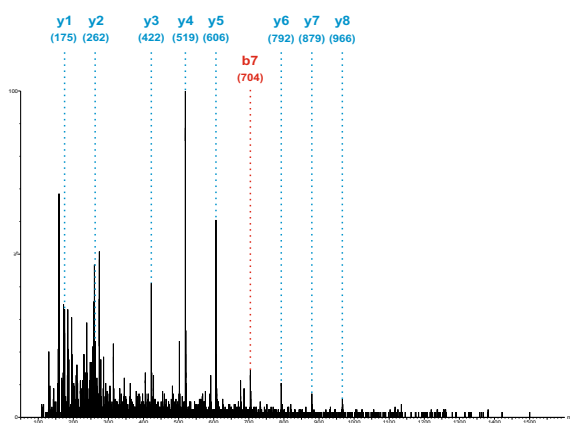

Supplement: Supplemental Figure S5 [file mmc5.pdf]
