# Supplementary material for: The C-Mannosylome of Human Induced Pluripotent Stem Cells Implies a Role for ADAMTS16 C-Mannosylation in Eye Development
Source: Mol Cell Proteomics. 2021 May 8;20:100092. doi: 10.1016/j.mcpro.2021.100092 (PMC8256286; doi:10.1016/j.mcpro.2021.100092)
Supplement: Supplemental Figure S4 [file mmc4.pdf]

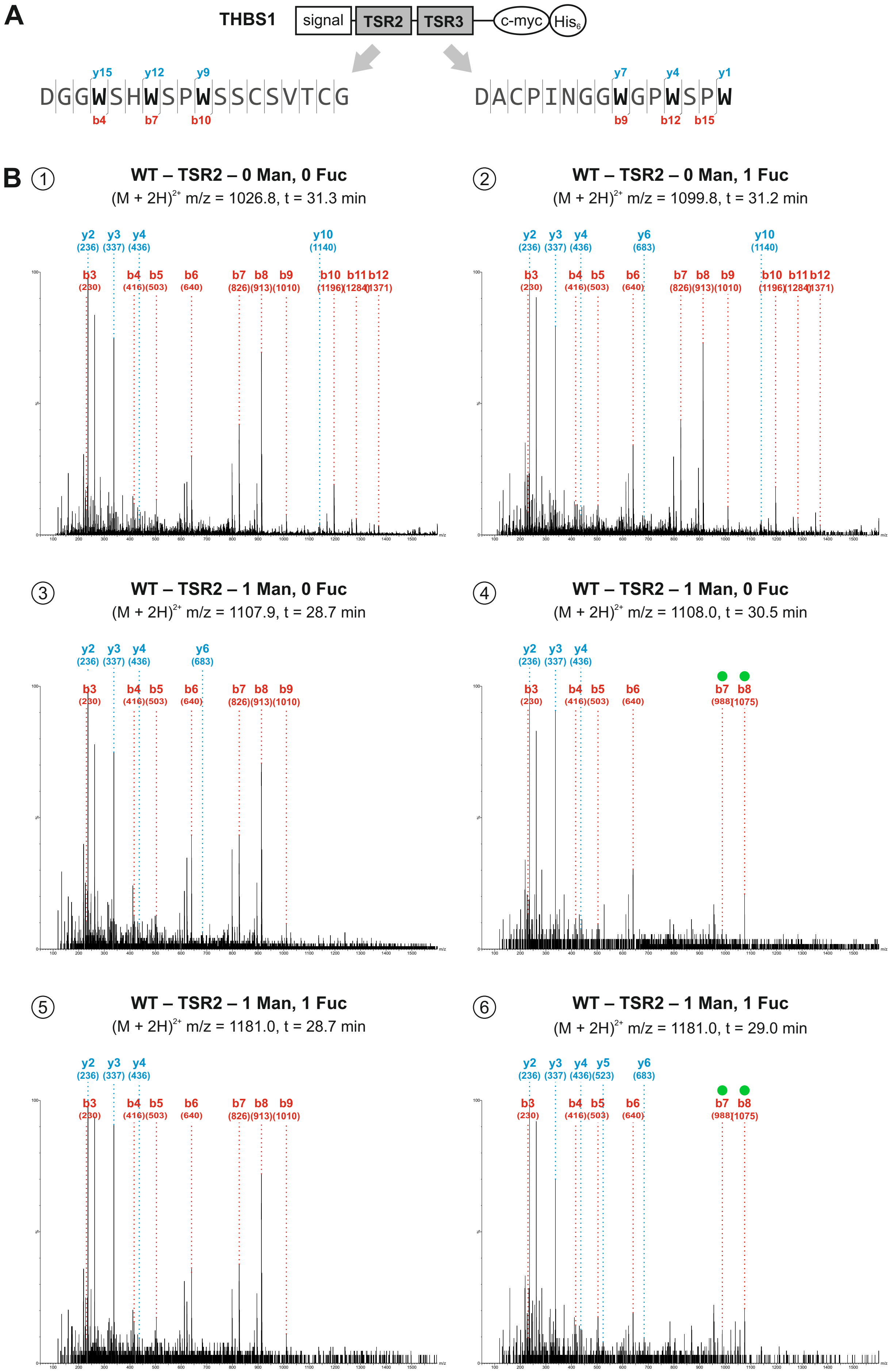
A
THBS1
signal
TSR2
TSR3
c-myc
His6
y15 y12 y9
DGGWSHWSPWSSSCSVTCG
b4 b7 b10
y7 y4 y1
DACPINGGWGPWSPW
b9 b12 b15
B
① WT – TSR2 – 0 Man, 0 Fuc
(M + 2H)2+ m/z = 1026.8, t = 31.3 min
② WT – TSR2 – 0 Man, 1 Fuc
(M + 2H)2+ m/z = 1099.8, t = 31.2 min
③ WT – TSR2 – 1 Man, 0 Fuc
(M + 2H)2+ m/z = 1107.9, t = 28.7 min
④ WT – TSR2 – 1 Man, 0 Fuc
(M + 2H)2+ m/z = 1108.0, t = 30.5 min
⑤ WT – TSR2 – 1 Man, 1 Fuc
(M + 2H)2+ m/z = 1181.0, t = 28.7 min
⑥ WT – TSR2 – 1 Man, 1 Fuc
(M + 2H)2+ m/z = 1181.0, t = 29.0 min
y2 (236) y3 (337) y4 (436) y5 (523) y6 (683) y10 (1140)
b3 (230) b4 (416) b5 (503) b6 (640) b7 (826) b8 (913) b9 (1010) b10 (1196) b11 (1284) b12 (1371)
b7 (988) b8 (1075)

⑦ **WT – TSR2 – 2 Man, 0 Fuc**

$(M + 2H)^{2+}$ m/z = 1188.9, t = 27.1 min

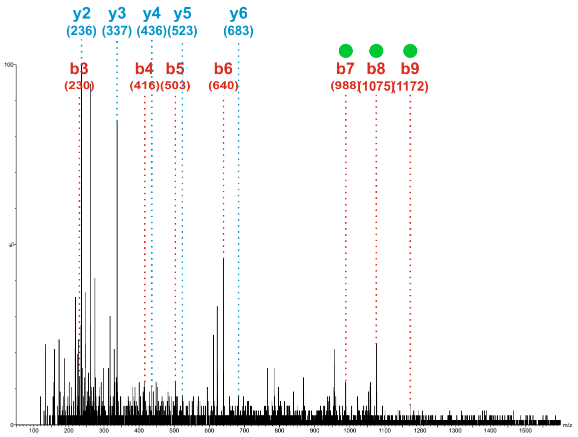

⑧ **WT – TSR2 – 2 Man, 0 Fuc**

$(M + 2H)^{2+}$ m/z = 1188.9, t = 28.1 min

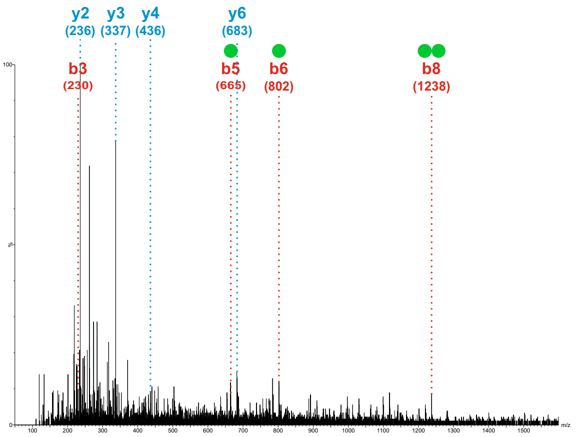

⑨ **WT – TSR2 – 2 Man, 1 Fuc**

$(M + 2H)^{2+}$ m/z = 1262.0, t = 27.0 min

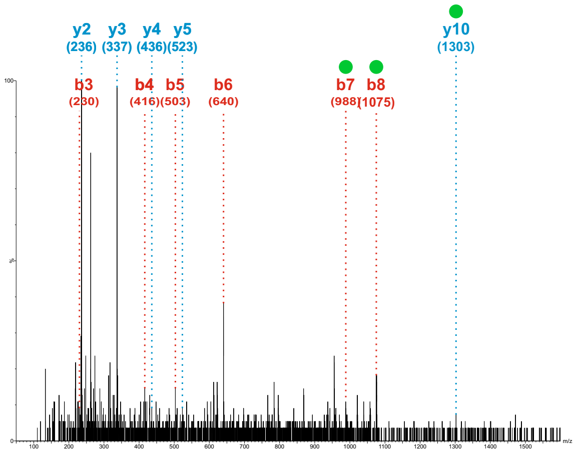

⑩ **WT – TSR2 – 2 Man, 1 Fuc**

$(M + 2H)^{2+}$ m/z = 1261.9, t = 28.2 min

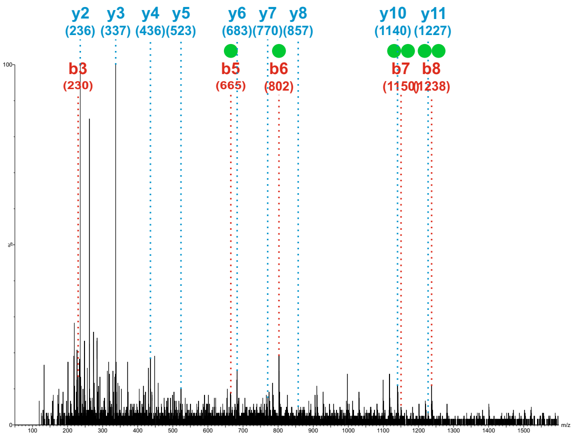

⑪ **WT – TSR2 – 1 Man, 1 Fuc-Glc**

$(M + 2H)^{2+}$ m/z = 1262.0, t = 29.5 min

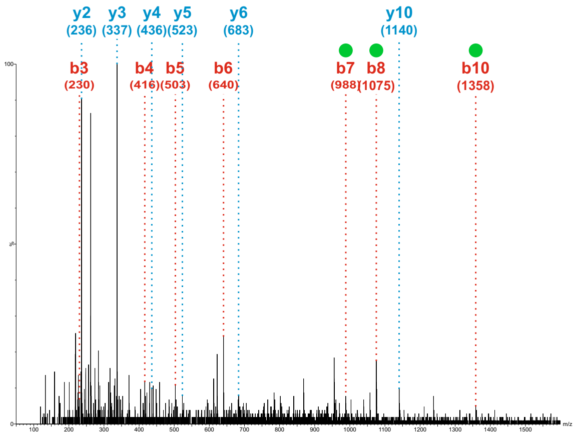

⑫ **WT – TSR2 – 3 Man, 1 Fuc**

$(M + 2H)^{2+}$ m/z = 1343.0, t = 25.6 min

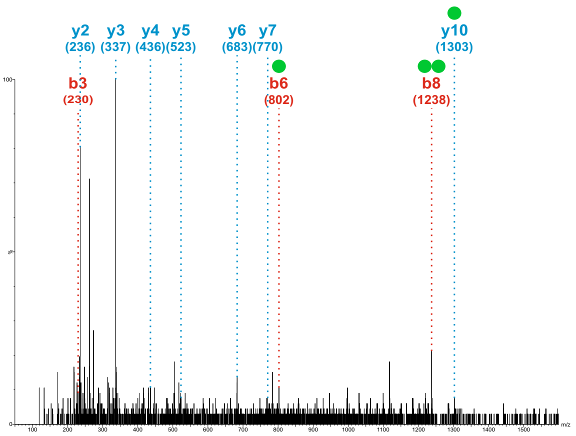

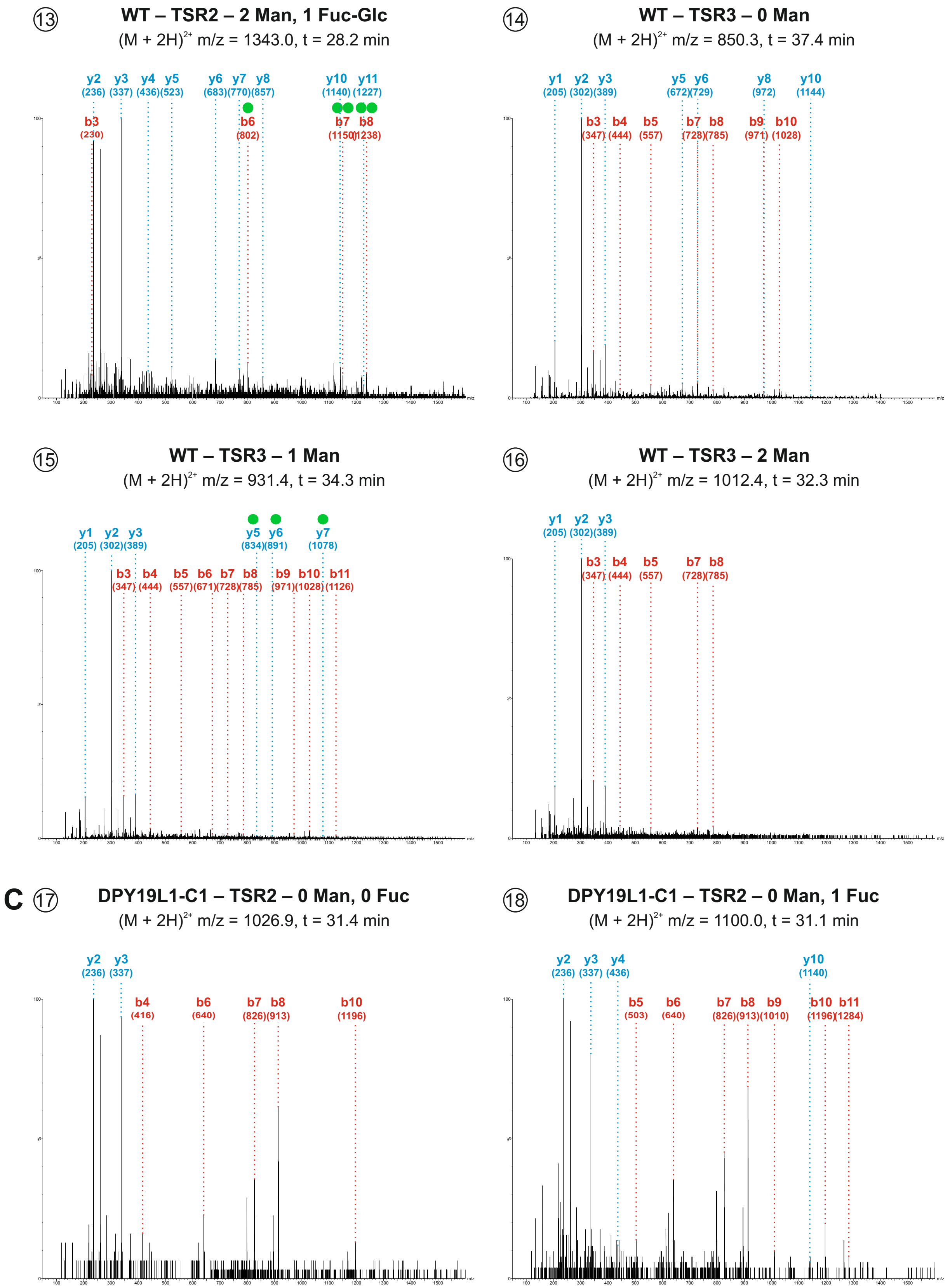
13
WT – TSR2 – 2 Man, 1 Fuc-Glc
(M + 2H)$^{2+}$ m/z = 1343.0, t = 28.2 min
y2 (236) y3 (337) y4 (436) y5 (523) y6 (683) y7 (770) y8 (857) y10 (1140) y11 (1227)
b3 (230) b6 (802) b7 (1150) b8 (1238)
14
WT – TSR3 – 0 Man
(M + 2H)$^{2+}$ m/z = 850.3, t = 37.4 min
y1 (205) y2 (302) y3 (389) y5 (672) y6 (729) y8 (972) y10 (1144)
b3 (347) b4 (444) b5 (557) b7 (728) b8 (785) b9 (971) b10 (1028)
15
WT – TSR3 – 1 Man
(M + 2H)$^{2+}$ m/z = 931.4, t = 34.3 min
y1 (205) y2 (302) y3 (389) y5 (834) y6 (891) y7 (1078)
b3 (347) b4 (444) b5 (557) b6 (671) b7 (728) b8 (785) b9 (971) b10 (1028) b11 (1126)
16
WT – TSR3 – 2 Man
(M + 2H)$^{2+}$ m/z = 1012.4, t = 32.3 min
y1 (205) y2 (302) y3 (389)
b3 (347) b4 (444) b5 (557) b7 (728) b8 (785)
C
17
DPY19L1-C1 – TSR2 – 0 Man, 0 Fuc
(M + 2H)$^{2+}$ m/z = 1026.9, t = 31.4 min
y2 (236) y3 (337)
b4 (416) b6 (640) b7 (826) b8 (913) b10 (1196)
18
DPY19L1-C1 – TSR2 – 0 Man, 1 Fuc
(M + 2H)$^{2+}$ m/z = 1100.0, t = 31.1 min
y2 (236) y3 (337) y4 (436) y10 (1140)
b5 (503) b6 (640) b7 (826) b8 (913) b9 (1010) b10 (1196) b11 (1284)

⑲ **DPY19L1-C1 – TSR2 – 1 Man, 1 Fuc**

(M + 2H)$^{2+}$ m/z = 1180.9, t = 28.9 min

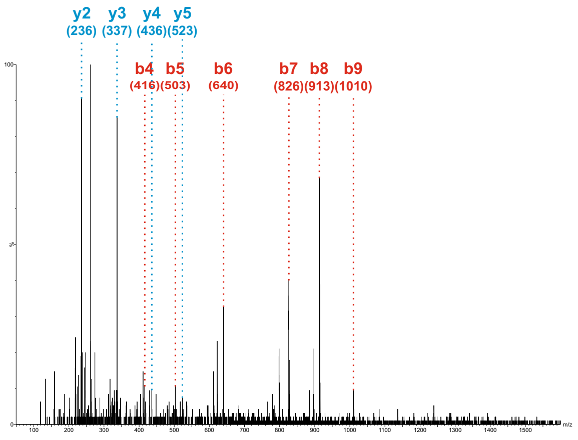

⑳ **DPY19L1-C1 – TSR2 – 0 Man, 1 Fuc-Glc**

(M + 2H)$^{2+}$ m/z = 1181.0, t = 30.6 min

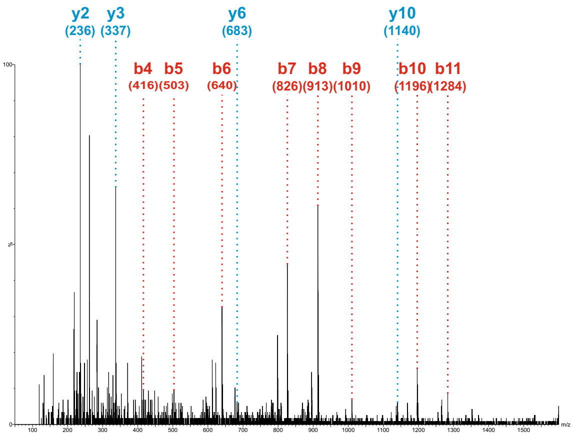

㉑ **DPY19L1-C1 – TSR2 – 1 Man, 1 Fuc-Glc**

(M + 2H)$^{2+}$ m/z = 1262.0, t = 28.6 min

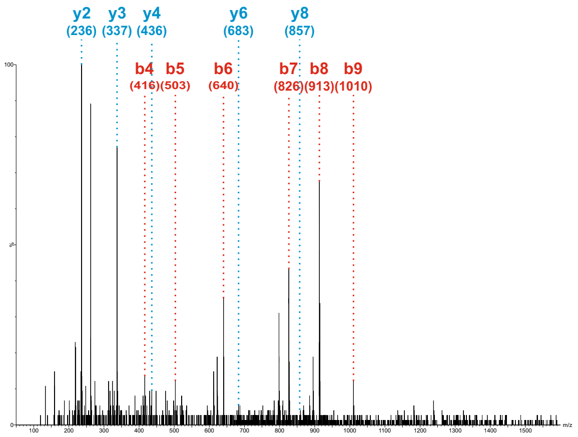

㉒ **DPY19L1-C1 – TSR3 – 0 Man**

(M + 2H)$^{2+}$ m/z = 850.3, t = 37.2 min

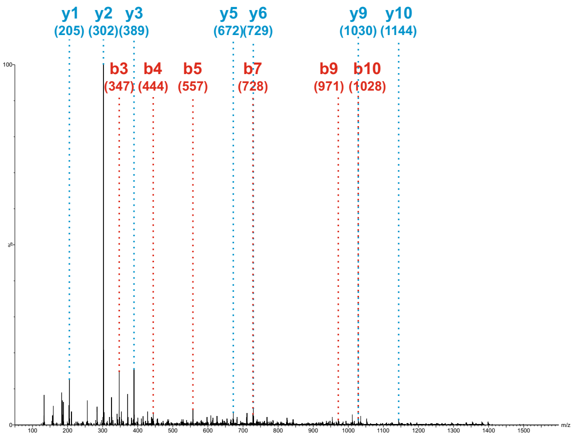

D ㉓ **DPY19L1-C2 – TSR2 – 0 Man, 1 Fuc**

(M + 2H)$^{2+}$ m/z = 1099.9, t = 31.3 min

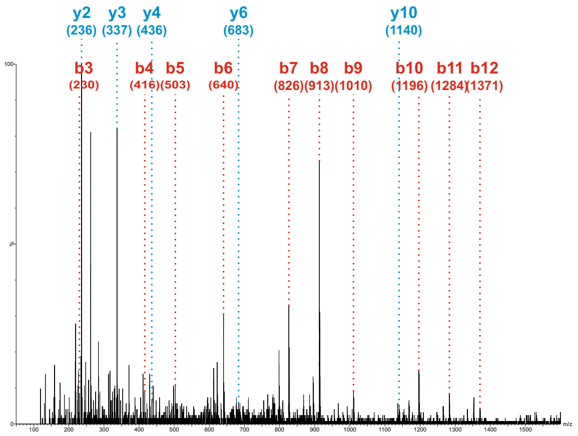

㉔ **DPY19L1-C2 – TSR2 – 1 Man, 1 Fuc**

(M + 2H)$^{2+}$ m/z = 1180.9, t = 29.0 min

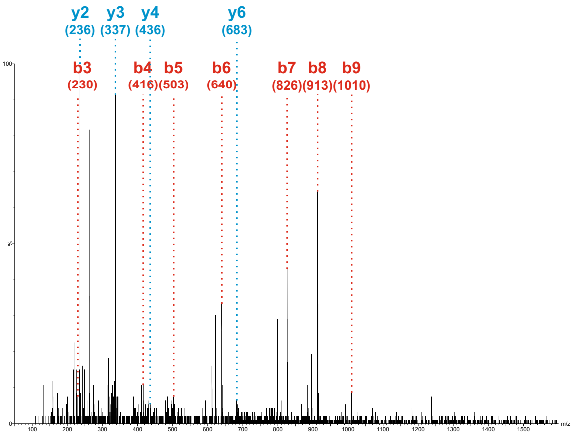

㉕ **DPY19L1-C2 – TSR2 – 0 Man, 1 Fuc-Glc**

(M + 2H)$^{2+}$ m/z = 1180.9, t = ~30.7 min

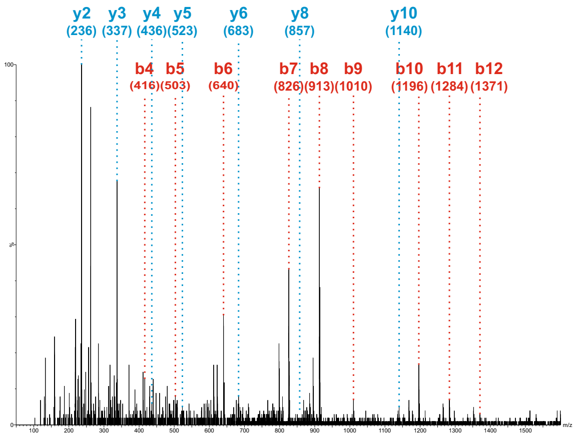

㉖ **DPY19L1-C2 – TSR2 – 1 Man, 1 Fuc-Glc**

(M + 2H)$^{2+}$ m/z = 1262.0, t = 28.6 min

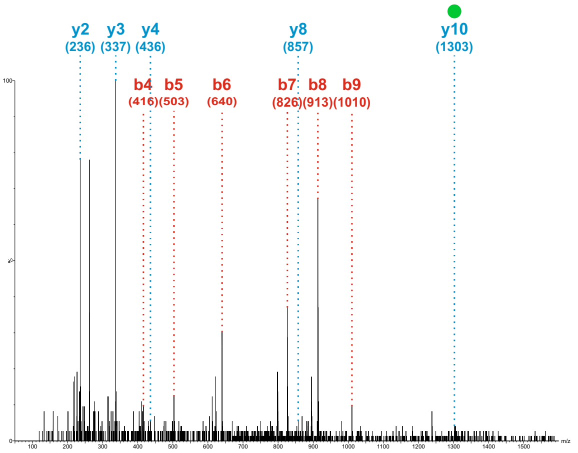

㉗ **DPY19L1-C2 – TSR3 – 0 Man**

(M + 2H)$^{2+}$ m/z = 850.4, t = 37.2 min

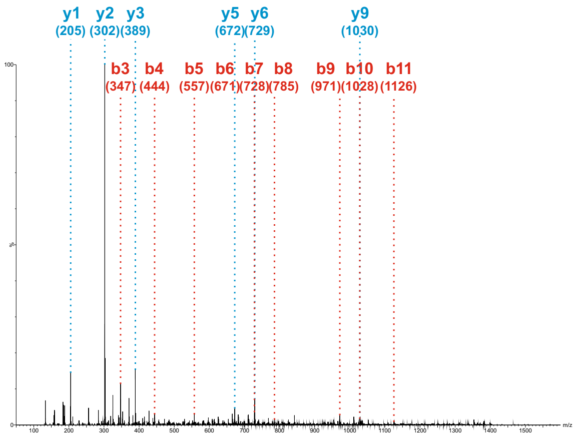

E ㉘ **DPY19L3-C1 – TSR2 – 0 Man, 1 Fuc-Glc**

(M + 2H)$^{2+}$ m/z = 1180.9, t = 31.0 min

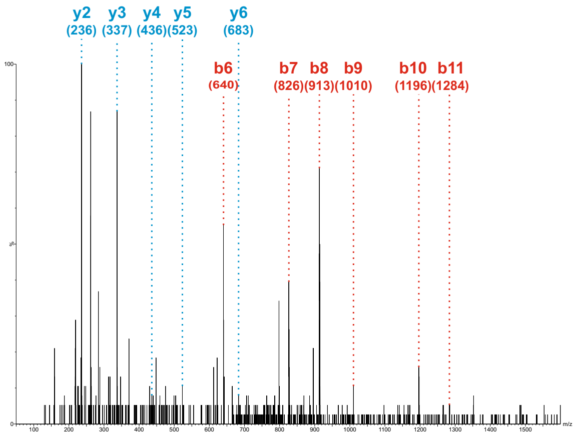

㉙ **DPY19L3-C1 – TSR2 – 2 Man, 1 Fuc**

(M + 2H)$^{2+}$ m/z = 1261.9, t = 28.2 min

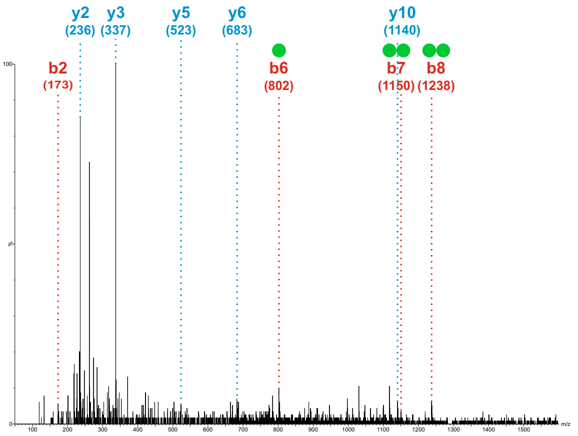

(30) **DPY19L3-C1 – TSR2 – 1 Man, 1 Fuc-Glc**

(M + 2H)$^{2+}$ m/z = 1261.9, t = 29.4 min

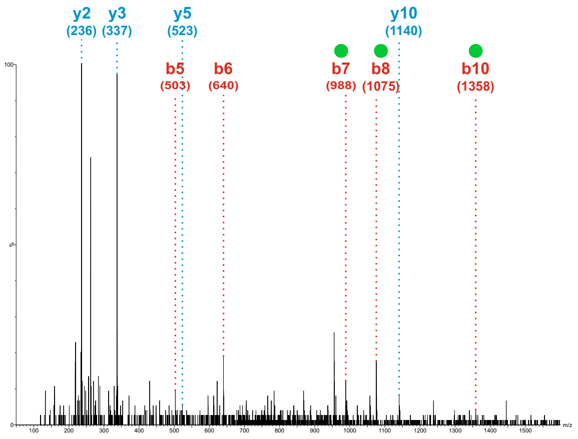

(31) **DPY19L3-C1 – TSR2 – 2 Man, 1 Fuc-Glc**

(M + 2H)$^{2+}$ m/z = 1342.9, t = 27.8 min

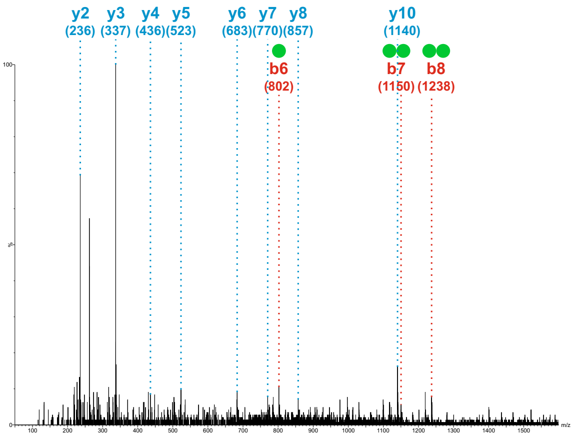

(32) **DPY19L3-C1 – TSR3 – 0 Man**

(M + 2H)$^{2+}$ m/z = 850.3, t = 37.4 min

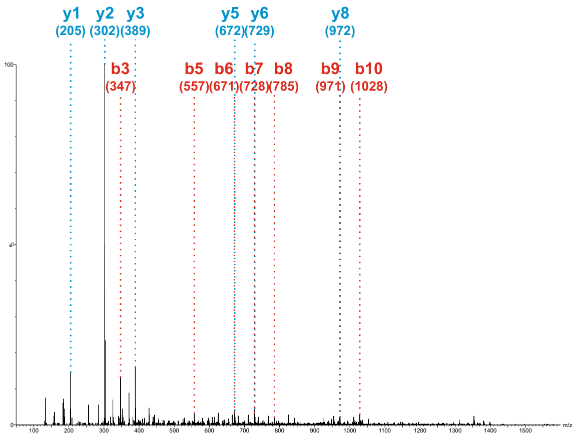

(33) **DPY19L3-C1 – TSR3 – 1 Man**

(M + 2H)$^{2+}$ m/z = 931.4, t = 34.6 min

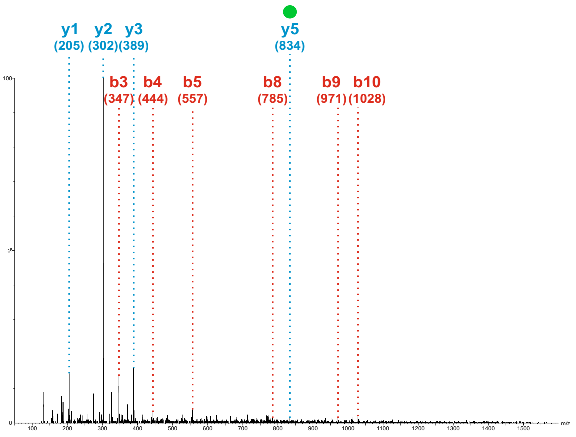

(34) **DPY19L3-C1 – TSR3 – 2 Man**

(M + 2H)$^{2+}$ m/z = 1012.4, t = 32.6 min

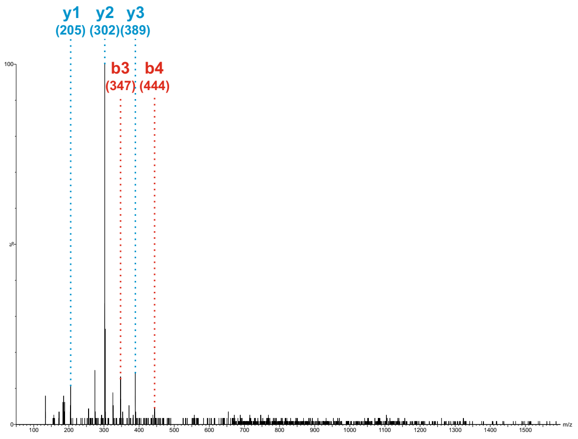

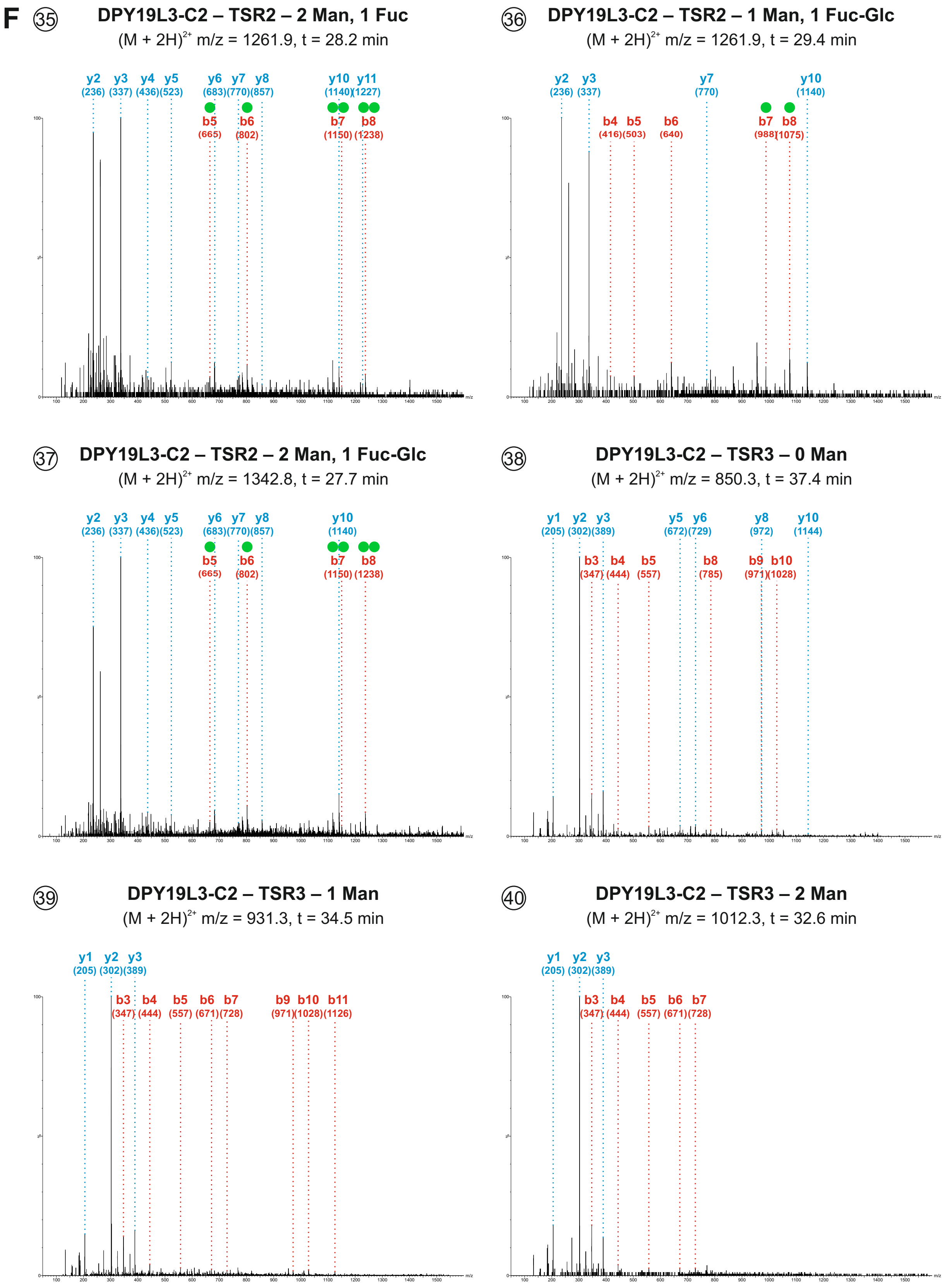
F
35 DPY19L3-C2 – TSR2 – 2 Man, 1 Fuc
(M + 2H)2+ m/z = 1261.9, t = 28.2 min
y2 (236) y3 (337) y4 (436) y5 (523) y6 (683) y7 (770) y8 (857) y10 (1140) y11 (1227)
b5 (665) b6 (802) b7 (1150) b8 (1238)
36 DPY19L3-C2 – TSR2 – 1 Man, 1 Fuc-Glc
(M + 2H)2+ m/z = 1261.9, t = 29.4 min
y2 (236) y3 (337) y7 (770) y10 (1140)
b4 (416) b5 (503) b6 (640) b7 (988) b8 (1075)
37 DPY19L3-C2 – TSR2 – 2 Man, 1 Fuc-Glc
(M + 2H)2+ m/z = 1342.8, t = 27.7 min
y2 (236) y3 (337) y4 (436) y5 (523) y6 (683) y7 (770) y8 (857) y10 (1140)
b5 (665) b6 (802) b7 (1150) b8 (1238)
38 DPY19L3-C2 – TSR3 – 0 Man
(M + 2H)2+ m/z = 850.3, t = 37.4 min
y1 (205) y2 (302) y3 (389) y5 (672) y6 (729) y8 (972) y10 (1144)
b3 (347) b4 (444) b5 (557) b8 (785) b9 (971) b10 (1028)
39 DPY19L3-C2 – TSR3 – 1 Man
(M + 2H)2+ m/z = 931.3, t = 34.5 min
y1 (205) y2 (302) y3 (389)
b3 (347) b4 (444) b5 (557) b6 (671) b7 (728) b9 (971) b10 (1028) b11 (1126)
40 DPY19L3-C2 – TSR3 – 2 Man
(M + 2H)2+ m/z = 1012.3, t = 32.6 min
y1 (205) y2 (302) y3 (389)
b3 (347) b4 (444) b5 (557) b6 (671) b7 (728)
