# Supplementary material for: The C-Mannosylome of Human Induced Pluripotent Stem Cells Implies a Role for ADAMTS16 C-Mannosylation in Eye Development
Source: Mol Cell Proteomics. 2021 May 8;20:100092. doi: 10.1016/j.mcpro.2021.100092 (PMC8256286; doi:10.1016/j.mcpro.2021.100092)
Supplement: Supplemental Table S2 [file mmc7.pdf]

		WT	DPY19L1				DPY19L3			
			Secretome		Transcriptome		Secretome		Transcriptome	
No.	Gene name	Mean LFQ	Fold change (log2)	p value	Fold change (log2)	p value	Fold change (log2)	p value	Fold change (log2)	p value
1	COL7A1[3]	5.94×10^{7}	-6.22	0.0010	-0.37	0.76	-6.84	0.0002	0.06	0.75
2	ANXA1	2.79×10^{8}	-3.07	0.0805	-0.73	0.86	-6.39	0.0029	3.06	0.00
3	IGFBP7	1.43×10^{8}	-3.17	0.0190	-0.14	0.68	-6.12	0.0008	0.33	0.46
4	SERPINE1	1.07×10^{9}	-2.57	0.0575	-0.01	0.29	-4.72	0.0028	0.98	0.59
5	WIF1	8.57×10^{6}	-3.52	0.0007	0.27	0.17	-3.02	0.0087	1.65	0.02
6	SERPINB9	1.97×10^{8}	-2.81	0.0580	-1.31	0.48	-3.53	0.0421	-1.15	0.15
7	S100A8	1.24×10^{7}	-3.83	0.0153	X	X	-1.92	0.1692	X	X
8	COL12A1	1.23×10^{8}	-2.17	0.0896	1.00	0.22	-3.51	0.0200	1.61	0.39
9	GDF3[2, 3]	5.42×10^{6}	-2.95	0.0316	-0.22	0.78	-2.21	0.1273	1.39	0.17
10	CER1	3.70×10^{7}	-2.92	0.0416	-2.45	0.60	-2.21	0.0777	0.38	0.07
11	GDF15[2]	6.14×10^{6}	-3.02	0.0228	0.01	1.00	-1.98	0.1249	-0.13	0.51
12	PCSK9	7.08×10^{8}	-2.33	0.0785	-0.21	0.40	-2.55	0.0389	1.11	0.20
13	COCH	3.98×10^{7}	-1.34	0.2830	-0.45	0.95	-3.48	0.0116	0.14	1.00
14	LGALS3	8.25×10^{6}	-1.75	0.1631	0.33	0.69	-2.86	0.0236	0.17	1.00
15	CTSL	1.17×10^{7}	-1.90	0.0665	0.24	0.56	-2.32	0.0276	0.38	0.68
16	SPP1	4.80×10^{8}	-1.28	0.1197	-1.09	0.86	-2.87	0.0024	0.58	0.06
17	ANXA2	7.66×10^{8}	-1.51	0.0348	0.33	0.20	-2.54	0.0063	1.05	0.11
18	COL6A2	2.05×10^{7}	-1.51	0.0506	-0.16	1.00	-2.48	0.0321	0.28	0.37
19	CALU	6.89×10^{7}	-2.11	0.0010	0.10	0.75	-1.84	0.0008	0.04	0.82
20	LEFTY1	4.74×10^{7}	-1.10	0.2173	-1.44	0.85	-2.76	0.0403	-0.34	0.26
21	LCP1	2.83×10^{8}	-0.35	0.9548	-0.20	0.98	-3.50	0.0223	1.06	1.00
22	CYR61	2.25×10^{7}	-0.95	0.1901	-1.34	0.54	-2.77	0.0226	-0.26	0.49
23	MCAM	5.49×10^{6}	-1.17	0.2315	-0.83	0.83	-2.40	0.0358	0.03	0.30
24	FETUB	1.34×10^{7}	-2.68	0.0174	-1.49	1.00	-0.88	0.2358	-0.73	1.00
25	HAPLN1[2]	2.88×10^{6}	-2.04	0.0150	-1.25	0.48	-1.46	0.0455	1.14	0.14
26	FGF19	4.62×10^{7}	-1.97	0.0345	-0.36	0.87	-1.49	0.1680	0.23	0.62
27	SDCBP	6.68×10^{6}	-2.20	0.0132	0.07	0.70	-1.24	0.1627	0.10	1.00
28	RNASET2	2.24×10^{7}	-0.72	0.2725	0.00	0.72	-2.71	0.0011	0.02	0.67
29	LAMC2	1.08×10^{7}	-0.47	0.4255	-0.66	0.90	-2.95	0.0068	0.26	0.61
30	LEFTY2	1.54×10^{8}	-0.53	0.3126	-1.37	1.00	-2.75	0.0371	0.58	0.02
31	LAMA5	7.45×10^{7}	-1.36	0.0021	-0.96	0.06	-1.73	0.0013	-0.35	0.37
32	ENPP2	1.02×10^{7}	-1.29	0.0537	-0.32	1.00	-1.69	0.0302	0.68	0.91
33	SERPINE2	2.69×10^{8}	-1.81	0.0423	-0.84	0.52	-1.15	0.1220	-0.02	1.00
34	SPARC	5.01×10^{9}	-1.59	0.0286	-0.04	0.94	-1.29	0.0427	0.11	0.79
35	CTSZ	3.52×10^{6}	-0.40	0.3998	-0.08	0.57	-2.47	0.0090	0.03	0.70
36	COL6A1	4.03×10^{8}	-1.11	0.0218	-0.53	0.76	-1.67	0.0014	0.02	0.85
37	FN1	1.07×10^{9}	-1.58	0.0291	0.21	1.00	-1.06	0.2003	0.53	0.56
38	PVR[3]	3.34×10^{6}	-0.47	0.4534	-0.29	0.94	-2.16	0.0189	0.02	0.91
39	CTGF	3.78×10^{8}	-0.55	0.4065	-0.90	0.23	-1.98	0.0100	-0.54	0.21
40	CD59	2.43×10^{7}	-0.94	0.2175	0.05	0.80	-1.59	0.0482	-0.21	0.39
41	COL1A2	1.42×10^{8}	-1.06	0.0712	0.33	1.00	-1.45	0.0478	0.36	0.39
42	OLFML3	2.53×10^{8}	-0.80	0.1733	0.15	0.88	-1.66	0.0036	-0.07	0.41
43	EMILIN2	2.50×10^{6}	-0.49	0.5301	0.32	0.64	-1.89	0.0208	0.64	0.62
44	PRDX4	7.04×10^{6}	-1.78	0.0190	0.19	0.53	-0.54	0.2465	0.35	0.21
45	FLNA	2.04×10^{6}	-1.36	0.0344	0.12	1.00	-0.95	0.1430	0.33	0.82
46	COL1A1	2.75×10^{8}	-0.74	0.2616	-1.02	0.05	-1.34	0.0497	-1.07	0.05
47	GPC4	6.77×10^{6}	-0.95	0.0848	-0.55	0.42	-1.12	0.0188	-0.52	0.33
48	MANF	6.59×10^{6}	-1.07	0.0464	0.35	0.55	-0.91	0.1377	0.34	0.86
49	MMP9	2.94×10^{7}	0.10	0.8001	-1.12	0.73	-1.92	0.0047	0.29	0.34
50	COL5A1	1.48×10^{6}	-1.02	0.0185	-0.57	0.52	-0.76	0.1689	-0.78	0.50
51	COL5A2	3.12×10^{7}	-0.09	0.8816	1.06	0.36	-1.54	0.0333	0.98	0.15
52	THBS1	2.16×10^{8}	-0.56	0.1257	-0.61	0.58	-1.05	0.0138	-0.34	0.06
53	LFNG	1.01×10^{7}	-0.70	0.1738	0.17	0.84	-0.90	0.0267	0.21	0.71
54	PCSK1N[3]	2.04×10^{6}	0.37	0.7404	-1.46	0.66	-1.85	0.0143	0.06	0.64
55	APOE	2.40×10^{9}	0.13	0.7750	0.11	0.57	-1.31	0.0427	-0.11	0.40
56	IGFBP6	6.05×10^{7}	-0.15	0.5344	1.64	0.33	-0.86	0.0306	0.81	0.67
57	CLIC1	1.45×10^{8}	0.85	0.0471	0.60	0.28	-0.15	0.5709	0.63	0.31
58	QSOX2	1.28×10^{6}	1.37	0.0375	-0.08	0.86	0.00	0.3951	0.09	0.82
59	PLTP	2.12×10^{8}	1.06	0.0409	0.06	0.58	0.46	0.2717	-0.11	0.43
60	ADAMTS8	3.85×10^{6}	0.88	0.0861	-0.04	0.55	0.90	0.0151	-0.01	0.98
61	HAPLN3	1.90×10^{7}	0.95	0.0072	0.47	0.60	1.15	0.0285	-0.15	0.15
62	GPC2	3.93×10^{6}	1.57	0.0326	0.30	0.59	0.60	0.4499	0.10	0.42
63	SMARCA4	1.64×10^{6}	1.75	0.0254	-0.25	0.44	0.96	0.4455	-0.10	0.66
64	OLFM1	4.20×10^{5}	1.91	0.0368	0.51	0.85	1.67	0.4686	0.27	0.66
65	SFRP1	3.48×10^{7}	1.62	0.0840	0.39	0.42	2.15	0.0282	-0.46	0.02
66	GNAS	3.51×10^{5}	0.94	0.1578	-0.05	0.57	2.94	0.0286	-0.03	0.26
67	CA11	8.05×10^{6}	2.31	0.0309	0.91	0.95	2.31	0.0234	-0.02	0.16
68	FGFBP3	1.60×10^{6}	2.76	0.0369	0.68	0.64	2.03	0.0467	0.22	0.49
69	CHGA	1.47×10^{8}	2.40	0.0025	1.44	0.68	2.69	0.0061	0.20	0.68
70	NCOA5[1]	3.22×10^{5}	3.14	0.1640	0.16	0.34	2.72	0.0493	0.65	0.23
71	BTBD17[1]	4.82×10^{5}	4.44	0.0007	1.05	0.49	5.18	0.0002	0.20	0.40
